# DHDK, a Plant-Derived Natural Small Molecule, Protects Against Doxorubicin-Induced Cardiotoxicity via the PPARG-CPT1B-FAO Axis

**DOI:** 10.3390/ph18111759

**Published:** 2025-11-18

**Authors:** Jing Hong, Fangyu Zhang, Ruizhen Zhang, Hongyang Fu, Dongang Shen, Xinyue Wang, Yuting Yang, Jiamei Wu, Lin Meng, Hongyang Lü, Xiwei Jiang, Yunli Zhao

**Affiliations:** 1 School of Pharmacy, Shenyang Pharmaceutical University, Shenyang 110016, China; 2 School of Medical Devices, Shenyang Pharmaceutical University, Shenyang 110016, China

**Keywords:** DHDK, cardioprotection, doxorubicin exposure, cardiotoxicity, PPARG, CPT1B, FAO

## Abstract

**Background:** Doxorubicin (DOX) is a highly effective chemotherapy drug, but its use is limited by dose-dependent cardiotoxicity, driving the search for protective natural products. Although the herb Viscum coloratum (Kom.) Nakai is known for its cardiovascular benefits, the cardioprotective effects and mechanisms of its isolated compound, DHDK, remain unexplored. **Methods:** The protective effect of DHDK was first evaluated in DOX-injured H9c2 cardiomyocytes. Subsequently, an integrated network toxicology (incorporating DOX-induced toxicity targets and relevant chronic disease pathways such as aging and lipid metabolism) and pharmacology (DHDK) approach identified core targets, which were then refined through Protein–Protein Interaction (PPI) analysis and molecular docking. The underlying mechanism was investigated using lipidomics and validated through a series of in vitro assays, including CCK-8, q-PCR, biochemical tests, and flow cytometry, as well as in an in vivo rat model. **Results:** DHDK significantly alleviated DOX-induced cardiomyocyte toxicity. Integrated analysis identified 56 intersecting targets, with PPARG confirmed as the primary target via PPI and molecular docking. Lipidomics revealed that DHDK potently attenuated DOX-induced accumulation of pathogenic lipids (e.g., fatty acids, ceramides). Mechanistically, DHDK activated PPARG, which in turn upregulated CPT1B, a key regulator of fatty acid β-oxidation (FAO). This enhanced cell viability, ATP production, and mitochondrial membrane potential while reducing oxidative stress. These protective effects, which were abolished by the inhibition of PPARG or CPT1B, were further validated in vivo. **Conclusion:** This study demonstrates that DHDK exerts its cardioprotective effect by activating the PPARG-CPT1B-FAO axis, effectively correcting lipid metabolic disorders. Given that lipid dysregulation is a hallmark of various internal metabolic diseases, DHDK may also hold therapeutic potential for other heart conditions driven by metabolic disturbances, such as diabetic cardiomyopathy, highlighting its broad relevance to the field of internal diseases.

## 1. Introduction

In clinical practice, anthracycline drugs, such as doxorubicin (DOX), are indispensable as first-line, broad-spectrum chemotherapeutic agents. They exert potent anti-tumor effects primarily by disrupting DNA replication [1,2] and are widely used to treat various solid tumors and hematological malignancies [3]. However, a major clinical challenge is their dose-dependent cardiotoxicity, which poses a significant threat to patient safety [4,5,6]. This risk is particularly critical during the perioperative period [7], where patients face hemodynamic stress, systemic inflammation, and endothelial dysfunction-factors that can synergistically exacerbate DOX-induced cardiac injury and compromise surgical outcomes.

Furthermore, the long-term cardiovascular sequelae in cancer survivors represent a serious concern, as chronic cardiac damage significantly impacts patients’ quality of life and long-term survival [8]. Currently, effective interventions for DOX-induced cardiotoxicity are extremely limited. Dexrazoxane (DXZ) is the only FDA-approved prophylactic agent; however, due to potential severe adverse effects, such as myelosuppression [9,10], its use is restricted, primarily offering some cardioprotection in specific high-dose chemotherapy regimens. Consequently, there is a significant lack of therapeutic options for protecting the heart during active chemotherapy and for mitigating chronic cardiotoxicity in long-term survivors. Therefore, developing novel cardioprotective agents that are highly effective, safe, and suitable for preventing both acute and chronic cardiotoxicity is an urgent public health priority.

The heart critically relies on lipid metabolism, particularly fatty acid β oxidation (FAO), to meet its high energy demands [11,12]. DOX exposure severely disrupts this myocardial metabolic homeostasis [13], manifesting as inhibited FAO, abnormal lipid accumulation, mitochondrial damage, and ultimately, a cellular energy crisis [14,15,16,17,18,19]. Critically, these metabolic disturbances are not only key drivers of acute cardiotoxicity but also, when persistent, accelerate the aging process of cardiomyocytes. This aging phenotype is characterized by mitochondrial dysfunction and chronic inflammation [20,21], which significantly increases the long-term risk of heart failure. Therefore, DOX-induced injury represents a complex progression of metabolic toxicity and chronic dysfunction [22,23].

Given this pathophysiology, targeting metabolic dysregulation presents a promising therapeutic strategy. (1*E*, 4*E*)-1,7-Bis(4-hydroxyphenyl)hepta-1,4-dien-3-one (DHDK) [24], a small-molecule compound isolated from *Viscum coloratum* (Kom.) Nakai (mistletoe)–a plant that has been reported to have cardiovascular protective properties [25]–emerges as a candidate. This study demonstrates that DHDK significantly alleviates DOX-induced chronic lipid metabolism disorders, cardiac aging, and overall cardiotoxicity. Mechanistically, we found that DHDK activates the nuclear receptor Peroxisome Proliferator Activated Receptor Gamma (PPARG). This activation effectively enhances fatty acid uptake and utilization in cardiomyocytes, promotes FAO via the Carnitine Palmitoyltransferase 1B (CPT1B) pathway, reduces toxic lipid deposition, and thereby restores myocardial energy homeostasis. Consequently, DHDK concurrently antagonizes lipid metabolism disorders and mitigates cardiac injury.

In summary, our findings identify DHDK as a promising plant-derived natural product that protects against DOX-induced cardiotoxicity by activating the PPARG-CPT1B-FAO axis, offering a novel strategy for preventing and treating DOX-related cardiac complications in both acute and chronic settings.

## 2. Results

### 2.1. DHDK Attenuates DOX-Induced Cardiotoxicity In Vitro

**Figure 1:**
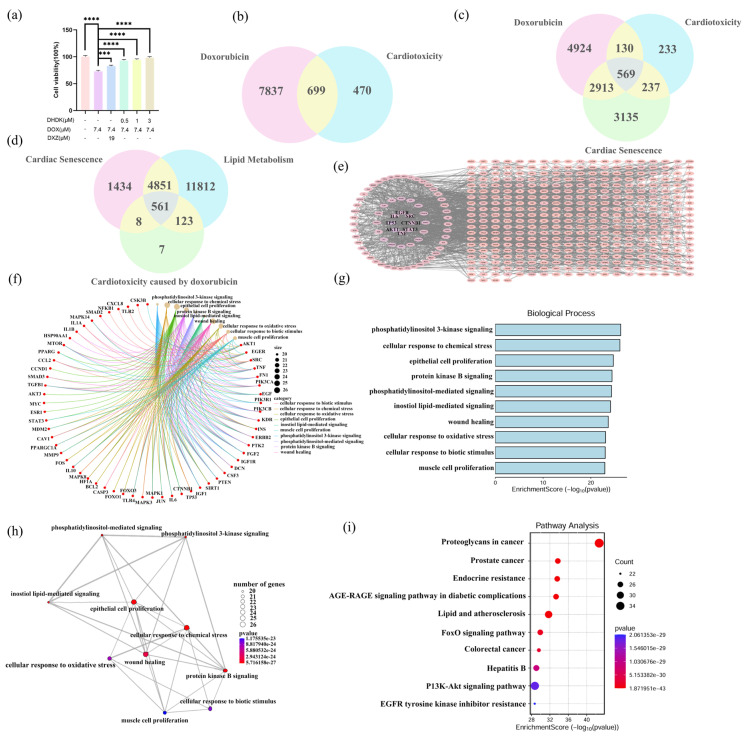
Integrated network toxicology analysis of DOX targets in cardiotoxicity and chronic disorders. CCK-8 results confirm that DHDK can alleviate cardiomyocyte damage caused by DOX (**a**), Venn diagram of DOX and potential targets of cardiotoxicity (**b**), Venn diagrams of DOX and cardiotoxicity, potential targets of cardiac aging (**c**), and Venn diagrams of potential targets of cardiotoxicity caused by DOX and cardiac aging and lipid metabolism (**d**). Protein–protein Interaction (PPI) plot (**e**), Gene Ontology (GO) results (**f**–**h**), and Kyoto Encyclopedia of Genes and Genomes (KEGG) enrichment analysis (**i**) results of DOX-induced cardiotoxicity and cardiac aging. Data are presented as mean ± SD (*n* = 3). Statistical significance was determined by Student’s *t*-test. *p < 0.05, **p < 0.01, ***p < 0.001, ****p < 0.0001, ns indicated no statistical significance.

We first evaluated the protective effect of DHDK against DOX-induced injury in cardiomyocytes. The CCK-8 assay demonstrated that DHDK significantly mitigated DOX-induced cytotoxicity in a concentration-dependent manner, with the most potent effect observed at a concentration of 3 μM (Figure 1a). At all three tested concentrations, DHDK treatment resulted in cell viability exceeding 90%, outperforming the positive control drug, DXZ, which achieved approximately 80% viability. These results indicate that DHDK possesses a marked cardioprotective function in vitro.

### 2.2. Identification of DOX Exposure Induced Acute and Chronic (Lipotoxicity, Cardiac Senescence) Cardiotoxicity Targets with Functional Enrichment Analysis

We investigated DOX-induced acute and chronic cardiotoxicity using network toxicology approaches. Shown in Figure 1b, integrated database analysis identified 8536 DOX-associated targets (List A) and 1169 cardiotoxicity-related targets (List B), yielding 699 putative targets of DOX-induced cardiotoxicity through intersection analysis (List E). Further tripartite analysis of Lists A, B, and 6854 cardiac senescence targets (List C) identified 569 intersecting targets that mediate both cardiotoxic and senescent responses to doxorubicin exposure (List F, Figure 1c). Cross-referencing these with 17,347 lipid metabolism targets (List D) obtained 561 (98.6%) targets, concurrently associated with lipid metabolism (List G), as shown in Figure 1d. This substantial overlap indicates that DOX-induced acute cardiotoxicity and chronic cardiac dysfunction likely converge on shared pathological mechanisms centered on lipid dysregulation.

The protein–protein interaction network construction of List F, using Search Tool for Recurring Instances of Neighbouring Genes (STRING) (interaction score > 0.900) and topological analysis via Cytoscape, revealed a robust interactome comprising 453 nodes with 1970 edges, exhibiting an average node degree of 8.8. This analysis identified 69 topologically central hub targets, including AKT serine/threonine kinase 1 (AKT1), Mitogen-Activated Protein Kinase 3 (MAPK3), PPARG, and Sirtuin1 (SIRT1), characterized by high centrality scores, as shown in Figure 1e.

Functional enrichment analysis demonstrated significant associations with cardioprotective mechanisms. Gene Ontology (GO) analysis revealed that DOX toxicity/senescence/lipotoxicity pathways were enriched in oxidative stress response, phosphatidylinositol-mediated signaling (particularly the PI3K-AKT cascade), and inositol lipid metabolism, with each pathway containing more than 20 validated targets. Notably, Phosphatidylinositol 3-kinase (PI3K) inhibition may reduce catalytic Phosphatidylinositol-3,4,5-trisphosphate (PIP3) production, disrupting Ca^2+^ homeostasis and mitochondrial function, thereby synergistically driving cardiotoxicity while accelerating senescence through compromised antioxidant capacity. Kyoto Encyclopedia of Genes and Genomes (KEGG) analysis further confirmed enrichment in lipid/atherosclerosis signaling and the PI3K-AKT pathways (20 genes/pathway), establishing lipid dysregulation as the central nexus connecting acute toxicity, chronic senescence, and lipotoxicity, as represented in Figure 1f–i.

Complementary toxicity prediction using ProTox-3.0 corroborated lipid metabolism disruption and membrane structural damage as primary mechanisms of DOX cardiotoxicity, shown in Supplementary Materials (Table S1). Specifically, DOX promotes mitochondrial phospholipid peroxidation, alters the composition of the sarcoplasmic reticulum membrane, suppresses fatty acid β-oxidation, and induces the accumulation of toxic lipid intermediates. These alterations collectively generate lipotoxic cardiomyopathy through Reactive Oxygen Species (ROS)-mediated damage and metabolic dysfunction, mechanistically unifying the observed network pharmacology findings with established pathological sequelae.

### 2.3. Identification of Potential Targets and Functional Enrichment Analysis of DHDK

**Figure 2:**
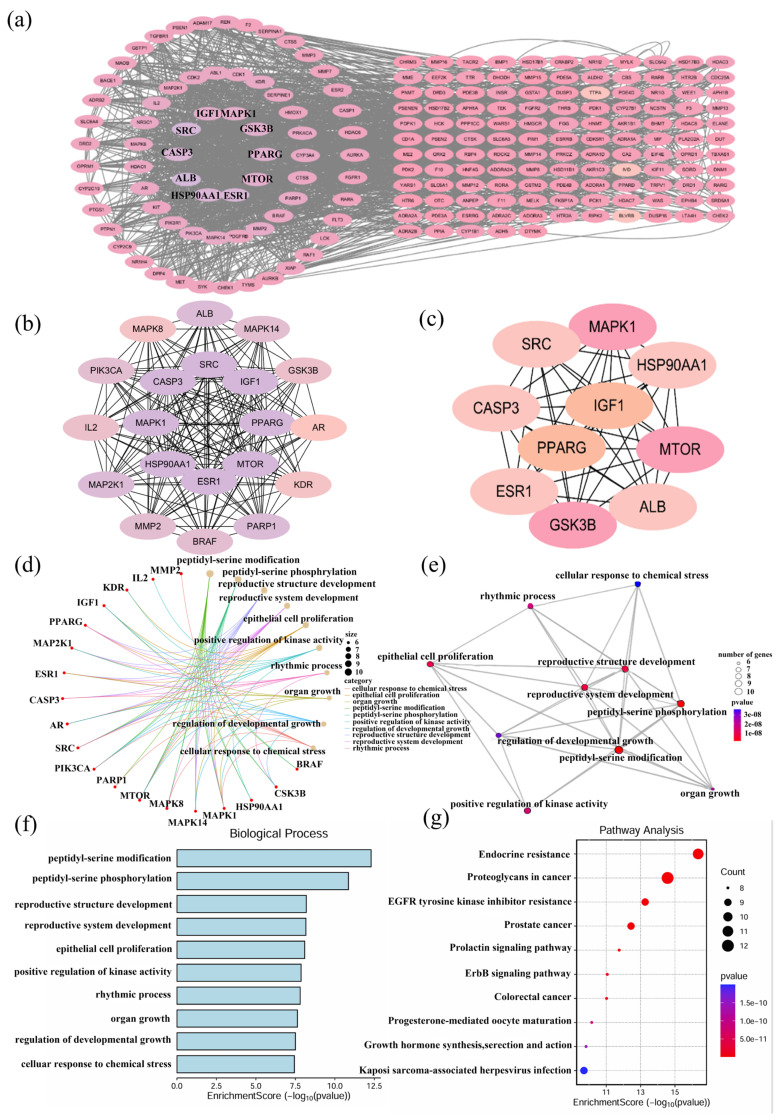
In vitro cardioprotective assessment of DHDK followed by network pharmacology analysis. PPI plot (**a**–**c**), GO results (**d**–**f**), and KEGG enrichment analysis (**g**) results of DHDK potential target.

Integrated screening through PharmMapper and SwissTargetPrediction identified 206 potential protein targets of DHDK. Subsequent protein–protein interaction network construction using STRING (visualized in Cytoscape 3.10.3) revealed a high-connectivity interactome comprising 196 nodes and 1801 edges. Topological analysis prioritized hub genes exhibiting the highest degree centrality, including PPARG, Estrogen receptor (ESR1), Insulin-like growth factor 1 (IGF1), Mitogen-activated protein kinase 1 (MAPK1), and Heat Shock Protein 90 Alpha Family Class A Member 1 (HSP90AA1), represented in Figure 2a–c.

Functional enrichment analysis revealed a significant association between DHDK and cardiovascular homeostasis mechanisms. GO terms highlighted regulation of lipid metabolism, oxidative stress response, angiogenesis modulation, and cellular senescence/apoptosis pathways. KEGG pathway analysis further revealed pronounced enrichment in cardioprotective signaling cascades, particularly Adenosine 5’-monophosphate (AMP)-activated protein kinase (AMPK) activation, sphingolipid metabolism, and Hypoxia inducible factor-1 (HIF-1)-mediated hypoxia response. Additional pathway associations encompassed lipid homeostasis, cellular senescence regulation, and longevity control networks, shown in Figure 2d–g.

Collectively, these hub targets position DHDK as a multi-mechanistic cardioprotective agent that concurrently modulates processes such as oxidative stress resolution, lipid metabolic reprogramming, and the balance between apoptosis and senescence, as well as maintaining vascular integrity.

This target profile indicates DHDK orchestrates cardiovascular protection through synergistic coordination of energy metabolism optimization, redox homeostasis preservation, and lipid signaling pathway regulation.

### 2.4. Hub Gene Identification and Enrichment Analysis of DOX Cardiotoxicity and DHDK-Mediated Cardio Protection

**Figure 3:**
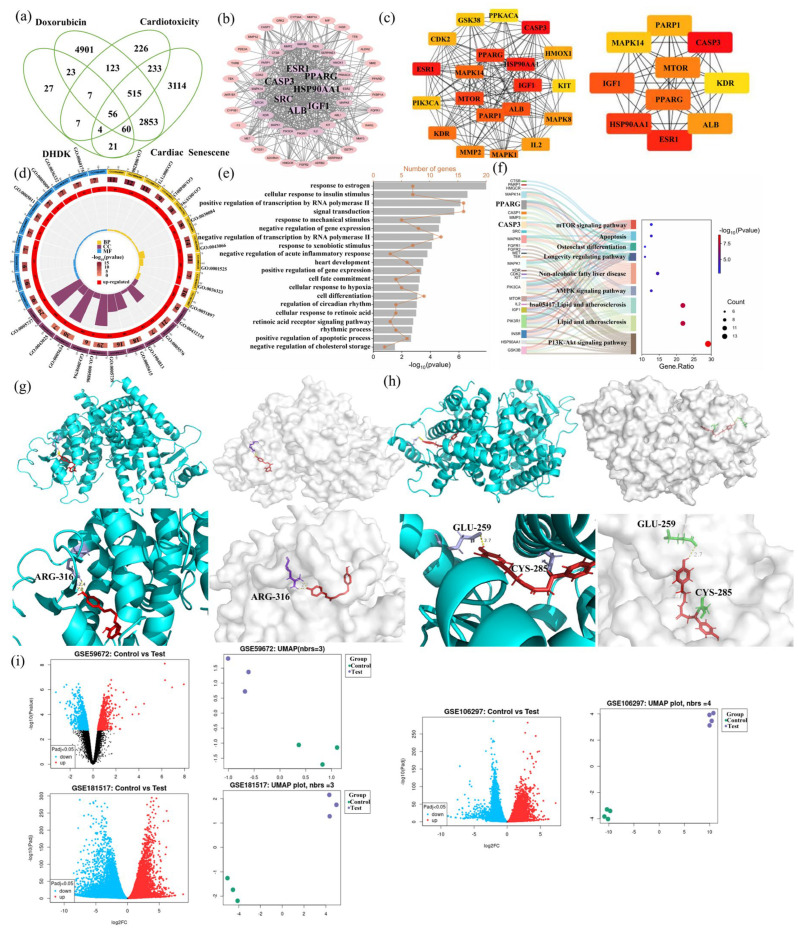
Integrated network toxicology-pharmacology analysis revealing PPARG as a convergent potential target in DOX-induced cardiotoxicity and DHDK-mediated cardioprotection. Venn diagram (**a**), PPI plot (**b**–**d**), GO enrichment analysis (**e**), and KEGG pathway enrichment analysis (**f**) of DHDK alleviation of DOX in cardiotoxicity and cardiac aging. Non-covalent molecular docking results (**g**) and covalent molecular docking results (**h**) of DHDK and PPARG, a potential alleviation of DOX-induced cardiac injury. The expression level of the potential target protein PPARG was obtained in the Gene Expression Omnibus (GEO) database before and after DOX-induced cardiac injury (**i**).

As shown in Figure 3a, integrated network toxicology and pharmacology analysis identified 56 overlapping hub genes functionally connecting DHDK’s activity to DOX cardiotoxicity mitigation. Protein–protein interaction analysis identified core targets that collectively orchestrate reactive oxygen species detoxification, intrinsic apoptosis suppression, and cellular senescence delay, as shown in Figure 3b–d. Functional enrichment, shown in Figure 3e,f, revealed significant enrichment (FDR < 0.05) in DOX-response pathways, including PI3K-Akt signaling pathway, apoptotic and senescence, lipid and atherosclerosis, and oxidative stress. These targets form functional nodes bridging DOX-induced pathology (ROS accumulation, metabolic disruption) and DHDK’s protection (lipid homeostasis restoration, antioxidant enzyme activation), demonstrating DHDK’s dual modulation of stress-response and metabolic stability networks to concurrently counteract acute cardiotoxicity and chronic cardiac dysfunction.

### 2.5. Molecular Docking Validation

We selected the top 7 PPI targets (Figure 3b) and listed them in Table 1. Non-covalent molecular docking demonstrated significant binding affinity between DHDK and core cardioprotective targets PPARG (ΔG = −9.7 kcal/mol), which is shown in Figure 3g, Albumin (ALB) (ΔG = −8.4 kcal/mol), and Caspase-3 (CASP3) (ΔG = −7.5 kcal/mol), driven primarily by hydrogen bonding with catalytic residues and complementary hydrophobic interactions within ligand-binding pockets. Further analysis revealed interactions involving van der Waals forces, π-sulfur interactions, and π-alkyl interactions. Given the presence of α, β-unsaturated ketone moieties in DHDK’s structure–electrophilic motifs capable of Michael addition with nucleophilic cysteine thiols–covalent docking simulations were performed (Figure 3h, Table 1 and Table S3). While ALB lacked accessible cysteine residues suitable for covalent modification, PPARG exhibited robust covalent adduct formation at Cys285 (ΔG = −7.5 kcal/mol), key residues that regulate receptor conformation and transcriptional activity. CASP3 showed weaker covalent binding potential at Cys163 (ΔG = −4.3 kcal/mol), a residue distal from its catalytic cleft. Comparative analysis revealed DHDK-PPARG as the optimal complex, which may adopt a unique dual-binding mechanism: initial high-affinity non-covalent recognition positions DHDK’s electrophilic carbonyl near Cys285, enabling subsequent irreversible covalent linkage. This synergistic binding mode anchors DHDK deep within PPARG’s ligand-binding domain in a stable agonist-like conformation, with key hydrogen bonds to Glu259 stabilizing the active state, which is critical for coactivator recruitment and the transactivation of cardioprotective gene networks. The molecular docking simulations (Figure 3g–h, Table 1 and Table S3) identified PPARG as the most promising high-affinity target for DHDK. PPARG may have a unique dual-binding mechanism. This computational finding was the direct rationale for focusing on PPARG. Hence, we hypothesized that DHDK acts as a PPARG activator.

### 2.6. The GEO Database Verified That the mRNA Expression Level of PPARG Was Significantly Affected by Doxorubicin

Transcriptomic validation using clinically relevant datasets from the Gene Expression Omnibus (GEO) database revealed consistent and significant downregulation of PPARG expression following DOX exposure. The GEO database verified that the mRNA expression level of PPARG was significantly affected by doxorubicin. Analysis of myocardial tissue from DOX-treated mice (GSE59672) demonstrated >2-fold reduction in PPARG mRNA levels compared to untreated controls (*p* < 0.001). This suppression was recapitulated in human cellular models: cardiomyocytes exposed to 1 μM DOX for 16 h (GSE106297) exhibited a 1.8-fold decrease in PPARG expression (*p* < 0.01), while an extended 24 h treatment (GSE181517) resulted in a 2.3-fold reduction (*p* < 0.0001), as shown in Figure 3i. The conserved downregulation across species and experimental systems indicates that PPARG pathway inhibition represents a fundamental mechanism underlying DOX cardiotoxicity. Importantly, this transcriptional repression aligns with our network pharmacology predictions and functional enrichment analyses, which positioned PPARG as a central regulator connecting lipid dysmetabolism, oxidative stress, and cellular senescence in DOX-damaged myocardium.

### 2.7. Lipidomic Alterations and Pathway Enrichment Analysis

**Figure 4:**
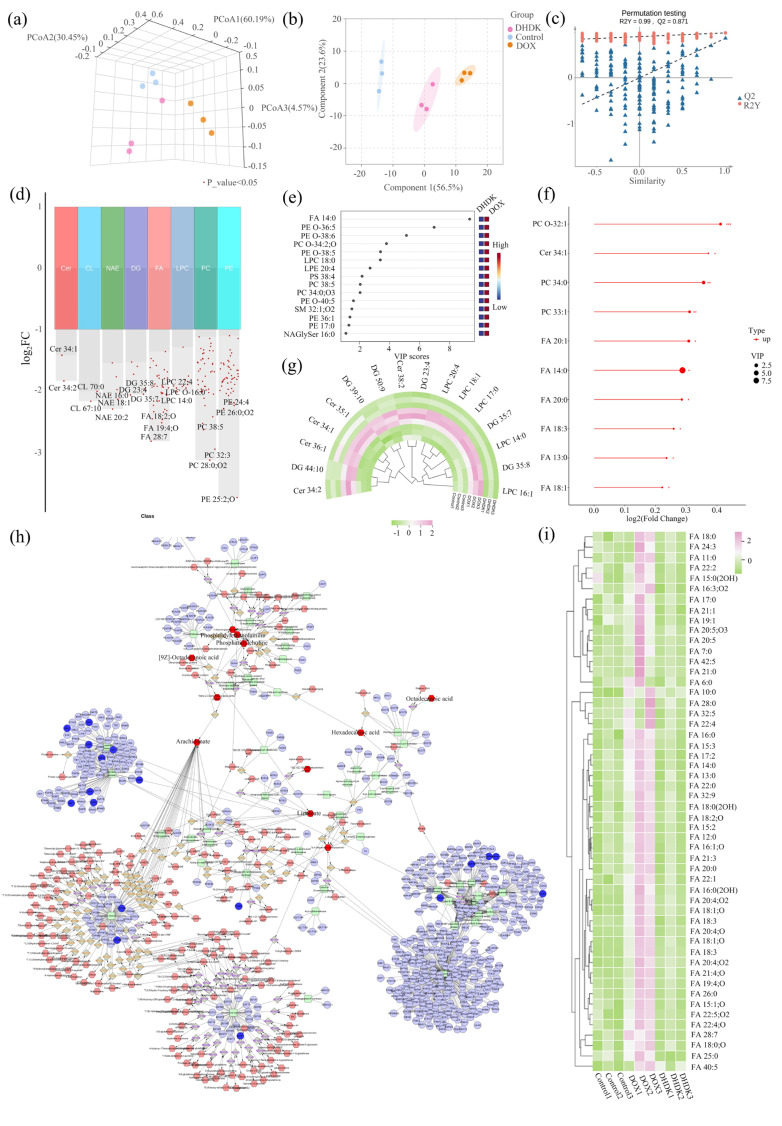
Integrated lipidomics and Hub Gene analysis reveal the potential mechanism of DHDK’s cardioprotection. The separation of each group in lipidomics was good, and the established model was reliable (**a**–**c**). Some differential lipid Log2FC values and Variable Importance in the Projection (VIP) values are shown (**d**). Heatmap visualization of representative lipid levels corrected for protein (**e**–**g**). The results of joint analysis of lipidomics, network pharmacology, and network toxicology, among which the arachidonic acid pathway is particularly critical (**h**). The content of fatty acids closely related to the arachidonic acid pathway in each group was focused on, and the results were visualized in a heatmap (**i**).

Lipidomic profiling revealed profound alterations in lipid species induced by DOX treatment, which are displayed in Figure 4a–c. KEGG pathway enrichment analysis indicated significant perturbations in arachidonic acid metabolism and glycerophospholipid metabolism, suggesting substantial disruption to cellular membrane architecture and bioactive lipid signaling during DOX-induced injury. This enrichment prompted a focused investigation into metabolic pathways governing membrane integrity and stress responses, as shown in Supplementary Materials (Table S2).

Compared to controls, DOX-exposed cells exhibited a marked accumulation of cytotoxic lipids, including the pro-apoptotic mediator ceramide (Cer), senescence-associated diacylglycerol (DG), and Lysophosphatidylcholine (LPC), which is closely related to phospholipid metabolism and the arachidonic acid pathway. Additionally, elevated fatty acid (FA) levels indicated dysregulated lipid storage or energy metabolism under DOX stress, as shown in Figure 4d–g.

Critically, DHDK co-treatment effectively reversed these lipid perturbations. DHDK significantly attenuated the accumulation of cytotoxic ceramides and senescence-promoting DG species. Notably, it restored mitochondrial membrane integrity by recovering cardiolipin (CL) levels—a vital phospholipid for cardiac mitochondrial function. These corrections collectively suggest DHDK mitigates membrane and organelle damage.

The coordinated lipidomic shifts–encompassing pro-apoptotic ceramides, senescence-linked DGs, membrane rigidity indicators, and disrupted energy reservoirs—underscore DOX’s multifaceted impact on lipid homeostasis. DHDK’s capacity to normalize these pathways highlights its potential role in preserving membrane dynamics, mitochondrial integrity, and metabolic balance during cardiotoxicity.

### 2.8. Combined Analysis of Lipodomics with Hub Genes

As shown in Figure 4h, lipidomic profiling revealed that DOX treatment induced pathological lipid accumulation, including pro-apoptotic ceramides and senescence-associated DG versus controls. Apart from that, the level of CL, which maintains mitochondrial homeostasis, decreased. DHDK co-treatment reversed these shifts, restoring protective lipid homeostasis and reducing the production of cytotoxic species. An integrated analysis of the hub gene network revealed a strong correlation between various lipid enrichment pathways, including fatty acid accumulation, the lipotoxicity-induced arachidonic acid pathway, and the expression patterns of core targets PPARG, SIRT1, and AKT1. It is worth noting that many of these dysregulated lipids are PPARG-regulated species, which are involved in oxidative stress, metabolic disorders, and other aspects, and are functionally related to the reduction of aging and apoptosis.

The lipidomics results showed that DHDK led to decreased fatty acid levels, Cer, etc. These data suggest that the cardio protection of DHDK may involve PPARG-mediated lipid reprogramming. Given PPARG’s role in lipid utilization, we propose that DHDK might activate PPARG to stimulate downstream fatty acid oxidation pathways, thereby alleviating DOX-induced lipotoxicity, cellular senescence, and cardiotoxicity, as illustrated in Figure 4i.

### 2.9. In Vitro Mechanism Validation of DHDK-Mediated Cardioprotection

**Figure 5:**
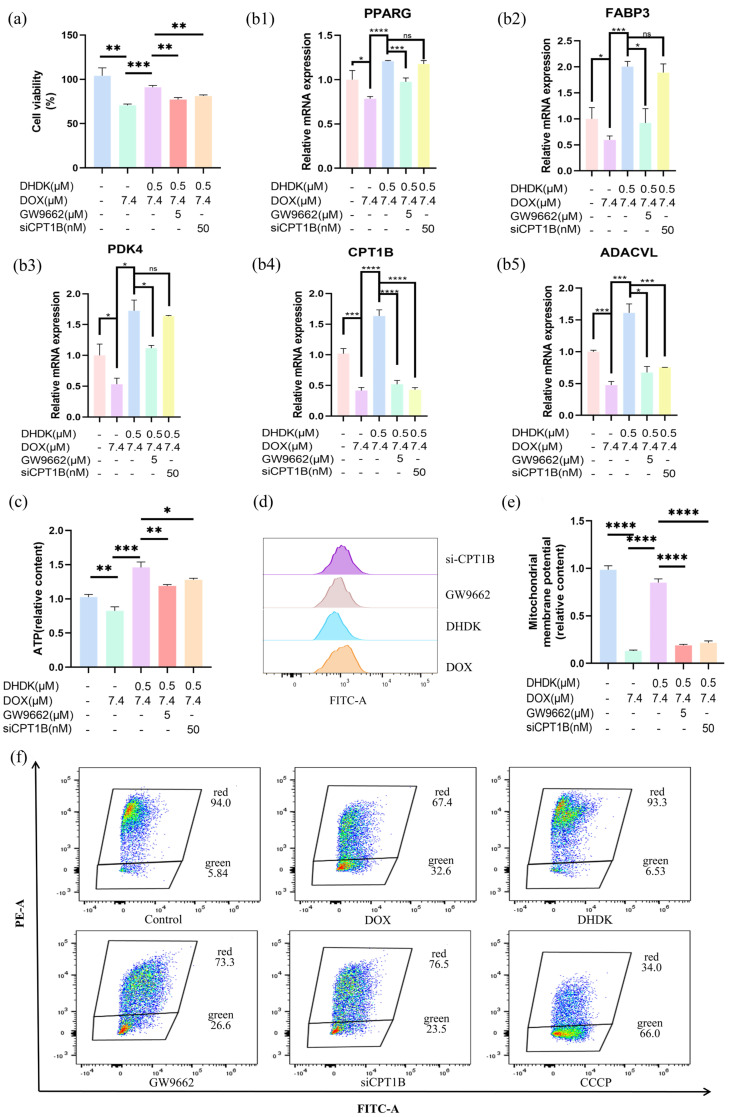
DHDK exerts cardioprotection by activating PPARG, which enhances fatty acid oxidation (in vitro validation). Cell viability assay (**a**), β-oxidation-related mRNA expression level (**b1**–**b5**), ATP content (**c**), ROS level (**d**), mitochondrial membrane potential (**e**,**f**) results. Data are presented as mean ± SD (n = 3). Statistical significance was determined by Student’s *t*-test. *p < 0.05, **p < 0.01, ***p < 0.001, ****p < 0.0001, ns indicated no statistical significance.

To functionally validate PPARG as the primary target of DHDK, we used the PPARγ antagonist GW9662 to assess cardiomyocyte viability. Our results (Figure 5a) showed that DHDK alleviated DOX-induced cardiotoxicity. However, the protective effect was significantly reduced when GW9662 was added. This key finding strongly suggests that DHDK’s cardioprotective effect is mediated by PPARG activation.

To further test whether the PPARG-CPT1B-FAO axis is necessary for DHDK’s effect, we assessed the role of the FAO enzyme CPT1B by siRNA knockdown and then measured cardiomyocyte viability. After CPT1B knockdown, DHDK’s protective effect was almost entirely lost (Figure 5a), demonstrating that disrupting this pathway abolishes the benefit. This result confirms that DHDK’s protection depends causally on an intact PPARG-CPT1B pathway.

We next studied the underlying mechanism. DOX injury profoundly suppressed PPARG expression (Figure 5b). As a result, key downstream targets linked to fatty acid oxidation (FAO) were downregulated. CPT1B enables mitochondrial fatty acid import. ACADVL mediates the initial β-oxidation step. FABP3 facilitates intracellular fatty acid transport. PDK4 regulates the metabolic shift from glucose to fatty acid use. DOX’s suppression of this program (Figure 5b) disrupts cardiac metabolic homeostasis. DHDK co-treatment restored PPARG and this gene network. This indicates rescue of the PPARG-mediated FAO pathway.

We confirmed the pathway’s causal role with loss-of-function experiments (Figure 5b). The PPARG antagonist GW9662, when co-administered, broadly reduced DHDK-induced upregulation of all five genes. This directly links PPARG inhibition to reduced gene expression and supports PPARG’s regulatory function. Knockdown of CPT1B by siRNA lowered only CPT1B and mainly affected ACADVL, its direct partner. The upstream regulator, PPARG, remained unchanged. This reveals CPT1B’s specific and essential pathway function.

This metabolic reprogramming reversed DOX-induced damage. Lipid metabolism normalized. ATP levels were restored (Figure 5c). Excessive ROS was reduced (Figure 5d), and ΔΨm recovered (Figure 5e,f). These effects depended on the PPARG-CPT1B/FAO axis. Disrupting this pathway with GW9662 or CPT1B knockdown weakened or abolished DHDK’s beneficial effects on gene expression, lipid toxicity, ATP, ROS, and ΔΨm (Figure 5b–f). The effects on ROS and ΔΨm were only partly reversed. This suggests minor auxiliary protective mechanisms may exist.

In summary, these results indicate that DHDK exerts cardioprotective effects by activating PPARG to promote fat oxidation.

### 2.10. In Vivo Validation of DHDK-Mediated Cardioprotection via PPARG

**Figure 6:**
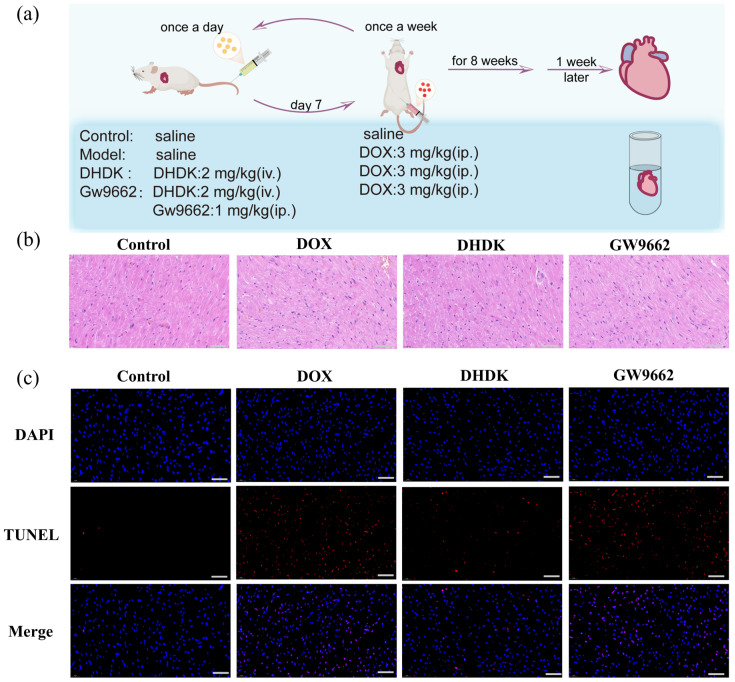
In Vivo Validation of PPARG as a key target for DHDK’s cardioprotection. Experimental dosing scheme for DHDK in animal study (**a**), HE staining results (**b**), and TUNEL staining results (**c**) of myocardial tissue. Scale bar  =  40 μm.

Intravenous injection of DHDK treatment (2 mg/kg/day) conferred robust cardioprotection against cumulative DOX-induced cardiotoxicity (3 mg/kg, once a week for 8 weeks) in rats, primarily through PPARG-dependent pathways. The DHDK dose (2 mg/kg/day, i.v.) was selected based on preliminary experiments that identified it as the lowest effective dose conferring significant cardioprotection. Histopathological analysis demonstrated that DHDK significantly ameliorated core pathological features of DOX injury. Furthermore, DHDK suppressed cellular damage by significantly reducing apoptosis. The animal experiment process is shown in Figure 6a.

The specificity of DHDK’s action to PPARG activation was unequivocally demonstrated by co-administration of the PPARγ (encoded by PPARG) antagonist GW9662 (1 mg/kg/day, i.p.). GW9662 significantly increased cardiotoxicity, demonstrating that DHDK’s cardioprotection is directly antagonized by PPARG blockade. This confirms the PPARG dependency as the primary mechanism of action, consistent with in vitro evidence of GW9662-mediated pathway inhibition (Figure 6b–c). Thus, DHDK’s in vivo efficacy in countering DOX-induced cardiotoxicity is predominantly mediated through the activation of PPARG.

## 3. Discussion

This study focuses on DOX-induced cardiotoxicity, showing it causes both acute injury and chronic, progressive damage marked by accelerated cardiac ageing. Using network toxicology and pharmacology analyses, we link anthracycline exposure to latent cardiac injury, including impaired fatty acid oxidation (FAO), toxic lipid accumulation, and overt cardiomyocyte senescence. These findings have serious clinical implications. Since anthracyclines are widely used in many tumors [26,27], including those with various doses [26,28,29], more patients are experiencing an irreversible decline in cardiac function years after therapy [6,27,30]. This process, driven by ongoing metabolic dysfunction and accelerated ageing, increases the risk of late-onset heart failure, lowering the quality of life and overall survival for cancer survivors. Therefore, therapeutic strategies must evolve to address both short and long-term DOX-induced cardiac damage.

We identify PPARG (Peroxisome proliferator-activated receptor gamma) as a critical node in this process. DOX (doxorubicin)-mediated downregulation of PPARG acts as an upstream trigger and directly suppresses a coordinated network of fatty acid utilization genes (including CPT1B, ACADVL, FABP3, and PDK4), which instigates an energy crisis and marked lipotoxicity. This metabolic disruption intensifies as mitochondrial dysfunction accompanies it, likely through impaired Peroxisome proliferator-activated receptor-γ coactivator-1α (PGC1α) [31] signalling, a protein that regulates genes involved in energy metabolism, and ultimately results in ATP (adenosine triphosphate) depletion, excessive ROS (reactive oxygen species) production, and collapse of the mitochondrial membrane potential (ΔΨm). Our integrated analyses based on network toxicology and pharmacology suggest that PPARG inactivation may link to senescence-associated pathways, in which NF-κB (nuclear factor kappa-light-chain-enhancer of activated B cells)-driven [32,33] inflammation can further amplify the secretion of Senescence-Associated Secretory Phenotype (SASP) factors [34], a collection of proteins secreted by senescent cells. This unifying hypothesis, which connects metabolic dysregulation, oxidative stress, and cellular senescence, offers a novel perspective for understanding DOX-induced chronic cardiotoxicity and underscores the therapeutic potential of targeting PPARG.

Our functional experiments further support this hypothesis. PPARG antagonism or CPT1B knockdown significantly attenuates DHDK’s cardioprotective effects, establishing the PPARG-CPT1B/FAO axis as the primary, non-redundant pathway mediating DHDK’s efficacy. DHDK orchestrates a comprehensive metabolic rescue by upregulating genes involved in fatty acid transport (FABP3), mitochondrial fatty acid import (CPT1B), β-oxidation (ACADVL), and metabolic substrate switching (PDK4). This transcriptional reprogramming normalises energy metabolism, restores ATP levels, quenches ROS, and stabilises ΔΨm, thereby reversing the core drivers of acute and chronic cardiotoxicity. Notably, when we disrupt the PPARG/CPT1B pathway, DHDK’s cardioprotective effects persist to some extent, suggesting a potential polypharmacological mode of action-a characteristic feature of many natural compounds. This ability to achieve synergistic efficacy through mild modulation of multiple targets, while avoiding the dose-limiting toxicities associated with potent single-target agonists, makes DHDK particularly suitable for long-term cardioprotection in cancer survivorship care.

DHDK offers superior therapeutic potential to conventional PPARG agonists. While synthetic ligands such as rosiglitazone show cardioprotective effects in preclinical studies, their clinical utility is limited by adverse effects, most notably fluid retention [35]. As a naturally derived compound, DHDK shows no significant toxicity in our previous assessments [36]. Furthermore, DHDK’s dual functionality-providing both cardioprotection and antitumor effects-suggests a transformative “dual-benefit” adjunct therapy [36]. Such an agent could protect the heart during chemotherapy and may improve the oncological efficacy of DOX, warranting further study.

We openly acknowledge a key limitation of this study: we currently rely on computational simulations to provide evidence of direct binding between DHDK and PPARG. We must pursue definitive validation through biophysical methods (e.g., CETSA) or structural biology approaches. Additionally, we must expand the evaluation of DHDK’s efficacy across diverse models of cardiotoxicity and other cardiovascular pathologies to fully delineate its therapeutic landscape.

In summary, this study provides a paradigm-shifting perspective on DOX-induced cardiotoxicity by elucidating its dual-risk profile, which encompasses not only the well-recognized acute injury but also the previously underappreciated chronic, progressive damage that manifests as accelerated cardiac ageing. We establish that PPARG signalling suppression acts as a central pathogenic hub, synchronously driving both injury modalities by disrupting lipid metabolic homeostasis and activating pro-senescent cascades. Importantly, we identified the natural compound DHDK as a novel PPARG activator and a desirable therapeutic candidate capable of counteracting these interconnected pathological processes. This work proposes a mechanistically grounded strategy to mitigate long-term cardiac sequelae in the growing population of cancer survivors, addresses a critical unmet need, and potentially reshapes the paradigm of supportive care in oncology.

## 4. Materials and Methods

### 4.1. Chemicals and Reagents

DHDK, a small-molecule compound, was isolated from *Viscum coloratum* (Kom.) Nakai (Batch number: 221201CP0669, originating from Liaoning, China), which was purchased from GuoDa drugstore (Shenyang, Liaoning). A voucher specimen (No. MS-20240806) has been deposited in the Department of Pharmaceutical Analysis, Shenyang Pharmaceutical University, Shenyang, China. The structural elucidation of DHDK is depicted in Supplementary Materials (Figure S1). Comprehensive structural characterization and purity analyses are presented in Supplementary Materials (Figure S2, Figure S3, and Figure S4, respectively). Finally, Supplementary Materials (Figures S5 and S6) provide a direct comparison with spectroscopic data previously reported in the literature for this compound [36]. The plant species was identified by Professor Yunli Zhao (Department of Pharmaceutical Analysis, Shenyang Pharmaceutical University, Shenyang, China) who has extensive research experience with this species. DHDK (purity 99.4%, yeild:0.0002%) was isolated and purified following previously established methods reported by the Department of Pharmaceutical Analysis at Shenyang Pharmaceutical University [24]. Comparative characterization data for DHDK are provided in the Supporting Information [36].

All commercial kits and reagents were obtained from the indicated suppliers. H9c2 rat cardiomyocytes and the CCK-8 kit (G4103) were purchased from Servicebio (Wuhan, China). Dimethyl sulfoxide (DMSO, 67-68-5, 99%) was obtained from Macklin (Shanghai, China); DOX (25316-40-9, 98%) and GW9662 (22978-25-2, 98%) were acquired from Bide (Shanghai, China); DXZ (24584-09-6, 98%) was supplied by InnoChem (Beijing, China). CPT1B knockdown siRNA was designed and synthesized by JTS Scientific (Shanghai, China). Kits for quantitative polymerase chain reaction (RC112, R433) were purchased from Vazyme (Nanjing, China) and Takara (Beijing, China, RR820A). Gene-specific primers were designed and synthesized by Sangon (Shanghai, China). The reactive oxygen species (ROS) detection kit (S0033S) was obtained from Beyotime (Shanghai, China); JC-1 (J8030) was sourced from Solarbio (Beijing, China); and the ATP determination kit (K2040) was acquired from APE × BIO (Houston, TX, USA).

### 4.2. Cell Availability

(1) H9c2(CRL-1446) rat cardiomyocytes (Servicebio, Wuhan, China) were seeded at 3000 cells per well in 96-well plates (Servicebio, Wuhan, China) and cultured for 24 h. Cell viability was assessed using the CCK-8 (Meilun, DaLian, China) assay, which involved adding 10 μL of CCK-8 reagent to each well, incubating at 37 °C for 1 h, and measuring the absorbance at 450 nm (HIPIE, Hangzhou, China). Survival rates were calculated by normalizing the optical density (OD) values to the DMSO (Maklin, Shanghai, China) control cells (100% viability). Each experiment was performed in triplicate, and three independent experiments were conducted to determine the IC_50_ values, which were then calculated using GraphPad Prism (version 8.0) (GraphPad Software, LLC, San Diego, CA, USA). To confirm that DHDK has a mitigating effect on cardiac injury caused by DOX, cell viability tests were conducted. The cells were then divided into six groups: 1) the Control group was cultured with complete medium and replaced with fresh complete medium once every 24 h. 2) the DOX (Bide, Shanghai, China) group pre-incubated with complete medium for 24 h followed by 7.4 μM DOX treatment for 24 h after medium removal; 3) the positive drug group (DXZ-DOX) incubated with 19 μM DXZ (InnoChem, Beijing, China) for 24 h, with subsequent medium replacement and 7.4 μM DOX exposure for 24 h, 4) DHDK group with different concentrations, in which cells were pre-incubated with 0.5, 1, 3 μM DHDK for 24 h before adding 7.4 μM DOX for an additional 24 h.

(2) In the study of the mechanism by which DHDK alleviates cardiac damage caused by DOX, H9c2 cells (Servicebio, Wuhan, China) were divided into five groups. The administration regimen for the Control group, Model group, and DHDK group is the same as described in (1). In the inhibitor GW9662 (Bide, Shanghai, China) group, cells were pretreated with GW9662 (5 μM) and DHDK (0.5 μM) for 24 h, followed by medium replacement and subsequent incubation with DOX (7.4 μM) for an additional 24 h. The CPT1B knockdown group was pretreated with DHDK for 24 h, followed by medium replacement and subsequent incubation with DOX (7.4 μM) for an additional 24 h. The CCK-8 detection method is the same as above.

CPT1B knockdown was performed using siRNA, as follows. siRNA was designed and synthesized by JTS Scientific (JTS, China) (Table S4). Cells were seeded in 6-well plates and cultured for 24 h to reach 50% confluence. The transfection operation was performed using Lipofectamine 3000 (Thermo Fisher Scientific, MA, USA) according to the manufacturer’s instructions. Briefly, siRNA was diluted in blank medium, and the mixture was incubated for 15 min at room temperature. Then, it was added to the cells. After 12 h, the supernatant was removed and the cells were incubated with fresh complete medium for an additional 48 h. After 48 h of transfection, cells were treated with drugs. Following treatment, cells were harvested or washed in situ for further analysis.

### 4.3. Data Collection and Target Extraction Involved Lipotoxicity, Cardiac Senescence and Cardiotoxicity of DOX

The potential target genes of DOX were collected from the PubChem and SwissTargetPrediction platforms (https://www.swisstargetprediction.ch, accessed on 17 May 2025) [37]. Data were also gathered from PharmMapper (https://www.lilab-ecust.cn/pharmmapper/index.html, accessed on 17 May 2025) [38,39,40], the Comparative Toxicogenomics Database (CTD) (https://ctdbase.org, accessed on 17 May 2025) [41] and Therapeutic Target Database (TTD) (https://db.idrblab.net/ttd, accessed on 17 May 2025) [42], generating a list of candidate genes (List A). The targets of cardiotoxicity and cardiac senescence were obtained from the Online Mendelian Inheritance in Man (OMIM) (https://www.omim.org/, accessed on 17 May 2025), TTD [42], and GeneCards (https://www.genecards.org/, accessed on 18 May 2025) [43,44]. The lists of candidate genes (Lists B and C) were generated, respectively. In addition, we obtained targets related to lipid metabolism from OMIM, TTD [42], CTD [41], and GeneCards [43,44], thereby compiling List D.

The intersection of List A and B was analyzed using Venny 2.1.0 (https://bioinfogp.cnb.csic.es/tools/venny/ accessed on 20 May 2025) to identify shared targets (List E), thereby achieving targets for DOX-induced cardiotoxicity. At the same time, we used ProTox 3.0- Prediction Of Toxicity Of Chemicals (https://tox.charite.de/protox3/index.php?site=home, accessed on 28 May 2025) [45,46] to predict the mechanism of DOX-induced cardiotoxicity (Table S1). The Venn diagram was also used to intersect List C, D, and E to associate the target of DOX-induced cardiotoxicity with lipid metabolism and cardiac senescence. Additionally, we identify the intersection of DOX, cardiotoxicity targets, and cardiac senescence targets for subsequent analysis (List F).

### 4.4. Network Construction and Core Target Identification Involved Lipotoxicity, Cardiac Senescence and Cardiotoxicity of DOX

The Venn diagram was used to intersect List A, B, and C to obtain targets related to cardiotoxicity and cardiac aging of DOX (List F). The protein–protein interaction network (PPI) of List F was constructed by STRING database (version 12.0, https://cn.string-db.org/, accessed on 1 June 2025) with Homo sapiens selected as the species of interest [47] and visualized in Cytoscape software (version 3.10.3) [48]. STRING was used to analyze the functional protein association network of the targets in List F, with a minimum required interaction score set at 0.900. DAVID (https://davidbioinformatics.nih.gov/conversion.jsp, accessed on 2 June 2025) [49,50] was employed to identify pathways and biological processes. Subsequently, GO Resource [51,52] and KEGG [53] enrichment analyses were used to reveal the connection between the core pathway and the core target.

### 4.5. DHDK Targets Prediction and Intersection with Cardiac Senescence and Cardiotoxicity

Potential targets of DHDK were predicted using SwissTargetPrediction PharmMapper [38,39,40] based on its chemical structure, yielding a candidate list (List H). The intersection of List A (DOX-related targets), B (cardiotoxicity-related targets), C (cardiac senescence-related targets), and H (DHDK-related targets) identified overlapping therapeutic targets (List I). The KEGG and GO treatment methods were the same as those described in Section 2.2, which involved lipotoxicity, Cardiac Senescence, and Cardiotoxicity of DOX.

### 4.6. Network Construction and Hub Target Identification of the Protective Effect of DHDK on Doxorubicin-Induced Lipotoxicity, Cardiac Senescence and Cardiotoxicity

The STRING database with Homo sapiens selected as the species of interest was used to retrieve the protein interaction and network generation of the targets in List I [47]. Subsequently, network visualization and deep network topology analysis were performed using Cytoscape 3.10.3, with the minimum required interaction score set to 0.90 [48].

The MCC algorithm in Cytoscape 3.10.3 was used to calculate the node ranking, and the top genes were derived as candidate Hub genes. The topological parameters were calculated using the NetworkAnalyzer tool, and the Hub genes were identified through degree sorting. Subsequently, GO [51,52] and KEGG [53] analyses were performed on the Hub gene to verify its biological significance.

### 4.7. Targets Validation via Molecular Docking

To verify the interaction between DHDK and the key targets (top 7 PPI targets (Figure 3b) identified in the above searches, the chemical structure of the small molecule drug DHDK was mapped in ChemDraw 3D and energy-minimized. The crystal structure of each protein was retrieved from the Protein Data Bank (PDB) (https://www.rcsb.org/, accessed on 5 June 2025), and the protein was docked with the small molecule drug DHDK using AutoDockTools [54] for non-covalent molecular docking to analyze the binding affinities and interaction modes.

The target protein was pretreated (i.e., deleting water and adding hydrogens, and computing charges) using AutoDockTools for non-covalent docking. Meanwhile, the small-molecule ligands were prepared, and both were converted to PDBQT format. The docking simulation in AutoDock Vina uses a grid centered on the active site (size covering the entire protein, exhaustiveness = 8) to rank the binding of each protein to the drug by affinity (ΔG < −7.5 kcal/mol). Visualization in PyMOL [55] was used to observe protein interactions with DHDK.

Regarding covalent docking for proteins with better non-covalent binding, the proteins were pretreated in Schrödinger 2023 [56,57,58,59,60,61]: filling in the missing side chains, assigning bond orders to all bonds in the structure, assigning bond orders to heteroatom groups using the Chemical Components Dictionary (CCD), replacing hydrogen atoms to repair hydrogen atoms in the original structure, repairing non-standard PDB atom names to achieve correct hydrogen bond assignment and optimizing hydrogen bonds, creating bonds for specific structural features, breaking bonds to metals, and correcting the formal charge of metals and their adjacent atoms as ionic bonds, then adding zero-order bonds between the metal and its ligands, creating disulfide bonds, finding two sulfur atoms within 3.2 Å distance and adding bonds between them, setting all selected heteroatom clusters in the pH range of 5.4–9.4 to be in a state of possible ionization and tautomerism, optimizing hydrogen bond assignment for proteins, and minimizing and removing water molecules.

Potential binding sites were identified using PyMOL and AutoDock, and the small molecule ligand DHDK was introduced and prepared. It was then covalently docked in the Glide module, with cysteine residues in the predicted site set as the center of the docking frame, and the reaction type defined as a Michael addition. The posture that ranked first by the docking score was chosen as the final covalent binding structure. The final structure is visualized in PyMOL.

### 4.8. Validate the Core Target from the GEO Database

The mRNA expression level data were retrieved from the Gene Expression Omnibus (GEO) database (http://www.ncbi.nlm.nih.gov/geo, accessed on 16 June 2025) for GSE59672 [62], GSE106297 [63], and GSE181517 [64]. These datasets comprise normal heart tissue versus DOX-treated heart tissue from three different platforms. GSE59672 was obtained from GPL1261 platforms [Mouse430_2] Affymetrix Mouse Genome 430 2.0 Array) and included 3 normal mouse heart tissues and 3 mouse heart tissues stimulated with DOX at 15 mg/kg for 5 days. GSE106297 were sourced from the GPL11154 platform (Illumina HiSeq 2000 (Homo sapiens)) and included 4 cells not treated with DOX and 4 cells treated with 1 μM DOX for 16 h. GSE181517 were sourced from the GPL18537 platform (Illumina NextSeq 500 (Homo sapiens)) and included 6 cells not treated with DOX and 6 cells treated with 1 μM DOX.

### 4.9. Lipodomics

H9c2 cells were seeded into 6-well plates (Servicebio, Wuhan, China), and the cells were divided into blank group, model group, and DHDK group, with drug treatment as in 2.7 (1). For lipid extraction, cells were washed three times with ice-cold (4 °C) PBS (Servicebio, Wuhan, China) to remove residual medium. Cell metabolism was quenched by the immediate addition of liquid nitrogen to culture dishes. After the liquid nitrogen had evaporated, 1 mL of 80% methanol (Sigma-Aldrich, Shanghai, China) was added to cover the cells. Cells were scraped using a steel scraper with unidirectional, steady motions to minimize mechanical stress and then transferred to 5 mL centrifuge tubes (Kirgen, Wuhan, China). Samples were flash-frozen in liquid nitrogen and stored at −80 °C. After processing all samples, three freeze–thaw cycles were performed. For each sample, 800 μL of PBS was added, incubated at −80 °C for 30 min, and then thawed at room temperature until the ice had melted. This process was repeated twice. Cell lysates were disrupted using an ultrasonic cell disruption system (XIAO MEI, Kunshan, China) on ice, followed by lipid extraction with 2 mL methyl tert-butyl ether (Damao, Tianjin, China) (MTBE; vortex 3 min, ice sonication 2 min). After 10 min incubation, samples were centrifuged (12,000 rpm, 4 °C, 5 min). Supernatants were transferred to new 1.5 mL tubes (Kirgen, Wuhan, China) (repeated twice), while the pellets were re-extracted with 1 mL MTBE (vortex (IKA, Germany) 1 min, stand 10 min, centrifugation (Cence, Hunan, China)). The combined supernatants were dried under a stream of nitrogen gas and stored at −80 °C.

For LC-MS analysis (Thermo Scientific, Waltham, MA, USA), dried lipid extracts were reconstituted in 150 μL of ice-cold isopropanol (Sigma-Aldrich, Shanghai, China) /methanol (1:1, *v*/*v*) with 2 min of vortexing. Samples were centrifuged (12,000 rpm, 4 °C, 10 min), and the supernatant was transferred and centrifuged again under identical conditions. The final supernatant was maintained at 4 °C, and 10 μL was injected for analysis.

Vanquish Horizon UHPLC separation (Thermo Scientific, Waltham, MA, USA) was employed with a column and a binary solvent system: Mobile phase A consisted of 10 mM ammonium acetate (Aladdin, Shanghai, China) and 0.1% formic acid (Sigma-Aldrich, Shanghai, China) in acetonitrile (Sigma-Aldrich, Shanghai, China)/water (60:40, *v*/*v*), while mobile phase B contained the same additives in isopropanol/acetonitrile (90:10, *v*/*v*). The gradient elution program was executed at a constant flow rate of 0.3 mL/min as follows: 85% A/15% B (0–3 min), linear transition to 70% A/30% B by 5 min, 35% A/65% B by 10 min, 20% A/80% B by 15 min, 10% A/90% B by 19 min, held until 23 min, followed by re-equilibration to initial conditions (85%A) at 24 min and maintained until 27 min.

Mass spectrometric detection was performed in positive/negative ion mode using full scan/data-dependent MS^2^ (dd-MS^2^) acquisition. Full scans covered the m/z range of 100–2000, with MS^2^ fragmentation implemented at collision energies of 20, 30, and 40 eV. All sample handling post-extraction was conducted at 4 °C to ensure lipid stability.

### 4.10. Combined Analysis of Lipodomics and Network Toxicology and Pharmacology

To investigate the therapeutic mechanism of DHDK through integrated lipidomics analysis, the identified core targets from PPI network analyses and KEGG/GO pathways were cross-referenced with significantly altered lipid species from lipidomic profiling (LC-MS data). Key lipid-metabolite-gene networks were constructed by mapping dysregulated lipids to their associated metabolic pathways and overlapping targets from prior network analyses. DHDK’s binding to core targets was correlated with lipidomic perturbations to identify potential functional axes. Cytoscape 3.10.3 was used to visualize integrated networks, where lipidomic changes, target proteins, and biological pathways were interconnected, revealing the coordinated effects of DHDK on lipid metabolism and signaling pathways in cardiovascular protection.

### 4.11. q-PCR

H9c2 cells were seeded in 6-well plates and subjected to the grouping/drug treatment regimen specified in Section 4.2, *Cell Availability* (2). Total RNA was isolated from treated cells using RNA extraction and separation kit (Vazyme, China, RC112), all RNA samples used exhibited an A260/A280 ratio of 1.8–2.0; all RNA samples used exhibited an A260/A280 ratio between 1.8 and 2.0. Followed by cDNA synthesis, total RNA was reverse-transcribed with the PrimeScript RT Master Mix (Vazyme, China, R433) according to the manufacturer’s protocol under the following conditions: 50 °C for 5 min and 85 °C for 5s. Gene-specific primers (Table S5) were designed and synthesized by Sangon. Then, primer specificity was validated using Primer Blast at NCBI. Q-PCR was performed on a QuantStudio 6 Flex system (Applied Biosystems) using TB Green Premix Ex Taq II (Takara, China, RR820A) under the following conditions: 95 °C for 30 s; 40 cycles of 95 °C for 5 s, 60 °C for 30 s; followed by melt curve analysis (65–95 °C, 0.5 °C/step). Each primer pair produced a single, distinct melting curve peak. Relative gene expression was normalized to β-Actin and calculated via the 2^−ΔΔCt^ method.

### 4.12. ROS Determination

Cell grouping and dosing regimens were applied prior to *4.11 q-PCR* analysis. Following treatment, cells were processed for ROS measurement. The DOX-containing culture medium was first aspirated, and cells were washed three times with phosphate-buffered saline (PBS). Cells were then incubated with the fluorescent ROS probe 2’,7’-Dichlorodihydrofluorescein diacetate (DCFH-DA) (Beyotime, Shanghai, China) (diluted according to the manufacturer’s instructions) for 30 min at 37 °C under standard culture conditions. After incubation, cells were washed three times with pre-warmed PBS under light-protected conditions to remove unincorporated probe. Subsequently, the detection was immediately performed using flow cytometry (Beckman Coulter, Brea, CA, USA).

### 4.13. Mitochondrial Membrane Potential Determination

A positive drug group was also added, and the other cell treatment methods were the same as those in Section 4.11 *q-PCR*. After stimulation with different drugs, the cells are washed twice with pre-warmed PBS solution. We then prepare a 2 μM fluorescent probe, JC-1 (Solarbio, Beijing, China), in blank medium and add it to the cells. The cells are incubated at 37 °C for 30 min in the dark. After incubation, the cells are washed twice with PBS to remove excess dye. Finally, detect mitochondrial membrane potential immediately with flow cytometry.

### 4.14. ATP Assay

The cell treatment is the same as in *4.11 q-PCR*. According to the instructions of APE × BIO (Houston, TX, USA), 200 μL of lysis buffer was added to each well in the six-well plate. After pipetting, centrifugation was performed at 12,000 g for 10 min at 4 °C, and the supernatant was collected for detection. At the same time, the ATP standard solution is sequentially diluted with lysis buffer for subsequent preparation of the standard curve. According to the number of samples and standards, the ATP detection working solution was configured, 100 μL was added to each detection well, placed at room temperature for 5 min, and then 20 μL of samples or standards was added to each well, mixed well, and the chemiluminescence signal was detected at 636 nm using a multi-function microplate reader (Envision, Sunderland, UK).

### 4.15. Animal Administration

Male Sprague Dawley rats (4–6 weeks old, weighing between 180–220 g, from Liaoning Changsheng Biotechnology Co., Ltd., China) were housed in a specific pathogen-free environment under a 12 h light/dark cycle with controlled temperature (22 ± 2 °C) and humidity (55–60%). The mice were randomly divided into 4 experimental groups (6 mice/group, 6 mice/cage, a total of 24 mice): (1) Control group: Daily tail vein injection of saline, followed by weekly intraperitoneal saline injection on Day 7. (2) DOX group: Daily tail vein injection of saline, with intraperitoneal DOX, (3 mg/kg) [65] administered weekly starting on Day 7. (3) DHDK + DOX group: Daily tail vein injection of DHDK (2 mg/kg), with weekly intraperitoneal DOX (3 mg/kg) starting on Day 7. (4) GW9662 + DHDK + DOX group: Daily tail vein co-injection of GW9662 (1 mg/kg) [66] and DHDK (2 mg/kg), with weekly intraperitoneal DOX (3 mg/kg) starting on Day 7. All treatments were continued for 8 weeks. One week after the final treatment, rats were euthanized under anesthesia, and hearts were immediately excised for further analysis.

All animal experiments were conducted in accordance with the relevant ethical regulations governing animal research. Ethical approval for this study (Ethical Committee No. SYXK(Liao)2021-0009) was provided by the Experimental Animal Ethical Committee of Shenyang Pharmaceutical University on 11 March 2024. This work has been reported in line with ARRIVE guidelines [67].

### 4.16. HE and TUNEL Staining of Heart Tissues

Cardiac tissues were fixed in 4% paraformaldehyde (Beyotime, China), embedded in paraffin, and sectioned at a thickness of 4 μm.

HE staining (Servicebio, China): Sections were dewaxed, hydrated, and stained with hematoxylin (5 min). They were then differentiated in 1% acid alcohol (5 s), blued in running water (15 min), counterstained with eosin (3 min), dehydrated, cleared in xylene, and mounted. Histopathological changes were examined under a light microscope (Olympus, Tokyo, Japan).

TUNEL assay (Servicebio, China): After dewaxing and hydration, sections were treated with proteinase K (20 μg/mL, 37 °C, 25 min) and permeabilized with 0.5% Triton X-100 (RT, 20 min). The TUNEL reaction mixture was applied (37 °C, 60 min, dark), followed by DAPI counterstaining (10 min, dark). Slides were mounted with an antifade medium. Apoptotic cells (red) and nuclei (blue) were visualized using a fluorescence microscope (Nikon, Tokyo, Japan).

### 4.17. Statistical Analysis

Data are presented as mean ± standard deviation. Difference comparisons were conducted by Student’s *t*-test (for two groups) using GraphPad Prism 8.0 software (GraphPad Software, LLC, San Diego, CA, USA). Statistical significance was set at *p* < 0.05.

## 5. Conclusions

This study demonstrates that DOX-induced acute and chronic cardiotoxicity is driven by disruption of lipid metabolic homeostasis. We identify the natural compound DHDK as a novel and potent cardioprotectant that directly targets PPARG. Molecular docking confirmed the stable binding of DHDK to PPARG through both non-covalent (−9.7 kcal/mol) and covalent (−7.5 kcal/mol) interactions, leading to the effective activation of the pathway. This activation restored lipid metabolic flux by enhancing fatty acid β-oxidation, which in turn reduced toxic lipid accumulation and alleviated subsequent cellular damage. By rectifying the core metabolic disturbance caused by DOX, DHDK represents a promising therapeutic strategy with broad applicability, offering protection against acute toxicity, long-term dysfunction, and other anthracycline-induced cardiac injuries.

## Figures and Tables

**Table 1:** Molecular docking results of DHDK with potential target proteins.

	Non-Covalent Molecule Docking (kcal/mol)	Covalent Molecule Docking (kcal/mol)
ESR1	−6.697	-
HSP90AA1	−7.058	-
IGF1	−6.081	-
CASP3	−7.543	−4.323
ALB	−8.398	-
SRC	−6.219	-
PPARG	−9.745	−7.495

## Data Availability

The raw data supporting the conclusions of this article will be made available by the authors on request.

## Supplementary Materials

The following supporting information can be downloaded at: https://www.mdpi.com/article/10.3390/ph18111759/s1, Table S1: ProTox-3.0–Prediction of TOXicity of chemicals; Table S2: Lipidomics pathway enrichment results; Table S3. List of comparative molecular docking with native ligand and DHDK. Table S4. List of siRNA (CPT1B) sequence. Table S5. List of sequence and other information of primers used in quantitative real-time PCR. Figures S1–S6: Characterization of DHDK and its comparison with previously reported data.

## Abbreviations

DOX	Doxorubicin
DHDK	(1E, 4E)-1, 7-Bis(4-hydroxyphenyl) hepta-1, 4-dien-3-one
PPI	Protein–Protein Interaction
PPARG	Peroxisome Proliferator Activated Receptor Gamma
CPT1B	Carnitine Palmitoyltransferase 1B
DXZ	Dexrazoxane
GEO	Gene Expression Omnibus
GW9662	PPARγ antagonist
VIP	Variable Importance in Projection
STRING	Search Tool for Recurring Instances of Neighbouring Genes
ATP	Adenosine triphosphate
FAO	Fatty Acid β-oxidation
AKT1	AKT serine/threonine kinase 1
MAPK3	Mitogen-Activated Protein Kinase 3
SIRT1	Sirtuin1
GO	Gene Ontology
KEGG	Kyoto Encyclopedia of Genes and Genomes
PI3K	Phosphatidylinositol 3-kinase
AKT	Protein Kinase B
PIP3	Phosphatidylinositol-3,4,5-trisphosphate
ESR1	Estrogen receptor
IGF1	Insulin-like growth factor 1
MAPK1	Mitogen-activated protein kinase 1
HSP90AA1	Heat Shock Protein 90 Alpha Family Class A Member 1
AMPK	Adenosine 5’-monophosphate (AMP)-activated protein kinase
HIF-1	Hypoxia inducible factor-1
ALB	Albumin
CASP3	Caspase-3
DG	Diacylglycerol
LPC	Lysophosphatidylcholine
CL	Cardiolipin
Cer	Ceramide
PPARγ	Peroxisome Proliferator-Activated Receptor Gamma
FABP3	Fatty Acid Binding Protein 3
PDK4	Pyruvate Dehydrogenase Kinase 4
ACADVL	Acyl-CoA Dehydrogenase Very Long Chain
ROS	Reactive Oxygen Species
SASP	Senescence-Associated Secretory Phenotype
PGC1α	Peroxisome proliferator-activated receptor-γcoactivator-1α
DMSO	Dimethyl Sulfoxide
OD	Optical Density
DCFH-DA	2’,7’-Dichlorodihydrofluorescein diacetate

## References

[1] Kciuk M., Gielecińska A., Mujwar S., Kołat D., Kałuzińska-Kołat Ż., Celik I., Kontek R. (2023). Doxorubicin-An Agent with Multiple Mechanisms of Anticancer Activity. Cells.

[2] Neefjes J., Gurova K., Sarthy J., Szabó G., Henikoff S. (2024). Chromatin as an old and new anticancer target. Trend Cancer.

[3] Damiani R.M., Moura D.J., Viau C.M., Caceres R.A., Henriques J.A.P., Saffi J. (2016). Pathways of cardiac toxicity: Comparison between chemotherapeutic drugs doxorubicin and mitoxantrone. Arch. Toxicol..

[4] Cardinale D., Colombo A., Lamantia G., Colombo N., Civelli M., De Giacomi G., Rubino M., Veglia F., Fiorentini C., Cipolla C.M. (2010). Anthracycline-induced cardiomyopathy: Clinical relevance and response to pharmacologic therapy. J. Am. Coll. Cardiol..

[5] Bostany G., Chen Y., Francisco L., Dai C., Meng Q., Sparks J., Sessions M., Nabell L., Stringer-Reasor E., Khoury K. (2025). Cardiac Dysfunction Among Breast Cancer Survivors: Role of Cardiotoxic Therapy and Cardiovascular Risk Factors. J. Clin. Oncol..

[6] Vo J.B., Ramin C., Veiga L.H.S., Brandt C., Curtis R.E., Bodelon C., Barac A., Roger V.L., Feigelson H.S., Buist D.S.M. (2024). Long-term cardiovascular disease risk after anthracycline and trastuzumab treatments in US breast cancer survivors. J. Natl. Cancer Inst..

[7] Tu Y., Zhou S., Wang H., Zhang P., Liu C., Zhu C., Yang C. (2024). Neoadjuvant chemotherapy and perioperative cardiotoxicity. J. Anesth. Transl. Med..

[8] Palmieri V., Vietri M.T., Montalto A., Montisci A., Donatelli F., Coscioni E., Napoli C. (2023). Cardiotoxicity, Cardioprotection, and Prognosis in Survivors of Anticancer Treatment Undergoing Cardiac Surgery: Unmet Needs. Cancers.

[9] Rahimi P., Barootkoob B., ElHashash A., Nair A. (2023). Efficacy of Dexrazoxane in Cardiac Protection in Pediatric Patients Treated with Anthracyclines. Cureus.

[10] Langer S.W. (2014). Dexrazoxane for the treatment of chemotherapy-related side effects. Cancer Manag. Res..

[11] Lee S.R., Mukae M., Jeong K.J., Park S.H., Shin H.J., Kim S.W., Won Y.S., Kwun H.J., Baek I.J., Hong E.J. (2023). PGRMC1 Ablation Protects from Energy-Starved Heart Failure by Promoting Fatty Acid/Pyruvate Oxidation. Cells.

[12] Grynberg A., Demaison L. (1996). Fatty acid oxidation in the heart. J. Cardiovasc. Pharmacol..

[13] Cabral R.P., Ribeiro A.P.D., Monte M.G., Fujimori A.S.S., Tonon C.R., Ferreira N.F., Zanatti S.G., Minicucci M.F., Zornoff L.A.M., Paiva S.A.R. (2024). Pera orange juice (*Citrus sinensis* L. Osbeck) alters lipid metabolism and attenuates oxidative stress in the heart and liver of rats treated with doxorubicin. Heliyon.

[14] Renu K., Vinayagam S., Madhyastha H., Madhyastha R., Maruyama M., Suman S., Arunachalam S., Vellingiri B., Valsala Gopalakrishnan A. (2022). Exploring the Pattern of Metabolic Alterations Causing Energy Imbalance via PPARα Dysregulation in Cardiac Muscle During Doxorubicin Treatment. Cardiovasc. Toxicol..

[15] Wang S., Zhang X., Hou Y., Zhang Y., Chen J., Gao S., Duan H., Gu S., Yu S., Cai Y. (2024). SIRT6 activates PPARα to improve doxorubicin-induced myocardial cell aging and damage. Chem. Biol. Interact..

[16] Xu Q., Wang X., Hu J., Wang Y., Lu S., Xiong J., Li H., Xiong N., Huang Y., Wang Y. (2025). Overexpression of hnRNPK and inhibition of cytoplasmic translocation ameliorate lipid disorder in doxorubicin-induced cardiomyopathy via PINK1/Parkin-mediated mitophagy. Free Radic. Biol. Med..

[17] Zhang S., Wei X., Zhang H., Wu Y., Jing J., Huang R., Zhou T., Hu J., Wu Y., Li Y. (2023). Doxorubicin downregulates autophagy to promote apoptosis-induced dilated cardiomyopathy via regulating the AMPK/mTOR pathway. Biomed. Pharmacother..

[18] Li W., Cao J., Wang X., Zhang Y., Sun Q., Jiang Y., Yao J., Li C., Wang Y., Wang W. (2021). Ferruginol Restores SIRT1-PGC-1α-Mediated Mitochondrial Biogenesis and Fatty Acid Oxidation for the Treatment of DOX-Induced Cardiotoxicity. Front. Pharmacol..

[19] Li H., Zhang M., Wang Y., Gong K., Yan T., Wang D., Meng X., Yang X., Chen Y., Han J. (2022). Daidzein alleviates doxorubicin-induced heart failure via the SIRT3/FOXO3a signaling pathway. Food Funct..

[20] Abdellatif M., Rainer P.P., Sedej S., Kroemer G. (2023). Hallmarks of cardiovascular ageing. Nat. Rev. Cardiol..

[21] Brown D.A., Perry J.B., Allen M.E., Sabbah H.N., Stauffer B.L., Shaikh S.R., Cleland J.G.F., Colucci W.S., Butler J., Voors A.A. (2017). Mitochondrial function as a therapeutic target in heart failure. Nat. Rev. Cardiol..

[22] Koleini N., Nickel B.E., Edel A.L., Fandrich R.R., Ravandi A., Kardami E. (2019). Oxidized phospholipids in Doxorubicin-induced cardiotoxicity. Chem. Biol. Interact..

[23] Saleh Y., Abdelkarim O., Herzallah K., Abela G.S. (2021). Anthracycline-induced cardiotoxicity: Mechanisms of action, incidence, risk factors, prevention, and treatment. Heart Fail. Rev..

[24] Zhao Y.L., Wang X.Y., Sun L.X., Fan R.H., Bi K.S., Yu Z.G. (2012). Cytotoxic constituents of Viscum coloratum. Z. Naturforsch C J. Biosci..

[25] Song C., Wei X.Y., Qiu Z.D., Gong L., Chen Z.Y., Ma Y., Shen Y., Zhao Y.J., Wang W.H., Lai C.J. (2021). Exploring the resources of the genus Viscum for potential therapeutic applications. J. Ethnopharmacol..

[26] Anthracycline Global Market Report 2025—By Drugs (Daunorubicin, Doxorubicin, Epirubicin, Idarubicin, Mitoxantrone, Val-rubicin), By Dosage (Powder, Capsule, Solution, Injection, Suspension, Other Dosages), By Application (Acute Lymphocytic Leukemia, Acute Myelogenous Leukemia, Hodgkin’s Lymphoma, Non-Hodgkin’s Lymphoma, Bladder Cancer, Breast Cancer, Other Metastatic Cancers), By End User (Hospitals, Homecare, Specialty Clinics, Other End Users)—Market Size, Trends, And Global Forecast 2025–2034. https://www.thebusinessresearchcompany.com/report/anthracycline-global-market-report.

[27] Nebigil C.G., Désaubry L. (2018). Updates in Anthracycline-Mediated Cardiotoxicity. Front. Pharmacol..

[28] Wang C., Zhang R., He J., Yu L., Li X., Zhang J., Li S., Zhang C., Kagan J.C., Karp J.M. (2023). Ultrasound-responsive low-dose doxorubicin liposomes trigger mitochondrial DNA release and activate cGAS-STING-mediated antitumour immunity. Nat. Commun..

[29] Perry J.M., Tao F., Roy A., Lin T., He X.C., Chen S., Lu X., Nemechek J., Ruan L., Yu X. (2020). Overcoming Wnt-β-catenin dependent anticancer therapy resistance in leukaemia stem cells. Nat. Cell Biol..

[30] Tanwar S.S., Dwivedi S., Khan S., Sharma S. (2025). Cardiomyopathies and a brief insight into DOX-induced cardiomyopathy. Egypt. Heart J..

[31] Ventura-Clapier R., Garnier A., Veksler V. (2008). Transcriptional control of mitochondrial biogenesis: The central role of PGC-1alpha. Cardiovasc. Res..

[32] Li Q., Sun J., Mohammadtursun N., Wu J., Dong J., Li L. (2019). Curcumin inhibits cigarette smoke-induced inflammation via modulating the PPARγ-NF-κB signaling pathway. Food Funct..

[33] Scirpo R., Fiorotto R., Villani A., Amenduni M., Spirli C., Strazzabosco M. (2015). Stimulation of nuclear receptor peroxisome proliferator-activated receptor-γ limits NF-κB-dependent inflammation in mouse cystic fibrosis biliary epithelium. Hepatology.

[34] Li T., Meng Y., Ding P., Wang H., Liu J., Xia C., Chen Y., Li J. (2023). Pathological implication of CaMKII in NF-κB pathway and SASP during cardiomyocytes senescence. Mech. Ageing Dev..

[35] Renauld S., Tremblay K., Ait-Benichou S., Simoneau-Roy M., Garneau H., Staub O., Chraïbi A. (2010). Stimulation of ENaC activity by rosiglitazone is PPARγ-dependent and correlates with SGK1 expression increase. J. Membr. Biol..

[36] Hong J., Meng L., Yu P., Zhou C., Zhang Z., Yu Z., Qin F., Zhao Y. (2020). Novel drug isolated from mistletoe (1E,4E)-1,7-bis(4-hydroxyphenyl)hepta-1,4-dien-3-one for potential treatment of various cancers: Synthesis, pharmacokinetics and pharmacodynamics. RSC Adv..

[37] Daina A., Michielin O., Zoete V. (2019). SwissTargetPrediction: Updated data and new features for efficient prediction of protein targets of small molecules. Nucleic Acids Res..

[38] Liu X., Ouyang S., Yu B., Liu Y., Huang K., Gong J., Zheng S., Li Z., Li H., Jiang H. (2010). PharmMapper server: A web server for potential drug target identification using pharmacophore mapping approach. Nucleic Acids Res..

[39] Wang X., Pan C., Gong J., Liu X., Li H. (2016). Enhancing the Enrichment of Pharmacophore-Based Target Prediction for the Polypharmacological Profiles of Drugs. J. Chem. Inf. Model..

[40] Wang X., Shen Y., Wang S., Li S., Zhang W., Liu X., Lai L., Pei J., Li H. (2017). PharmMapper 2017 update: A web server for potential drug target identification with a comprehensive target pharmacophore database. Nucleic Acids Res..

[41] Davis A.P., Wiegers T.C., Sciaky D., Barkalow F., Strong M., Wyatt B., Wiegers J., McMorran R., Abrar S., Mattingly C.J. (2025). Comparative Toxicogenomics Database’s 20th anniversary: Update 2025. Nucleic Acids Res..

[42] Zhou Y., Zhang Y., Lian X., Li F., Wang C., Zhu F., Qiu Y., Chen Y. (2022). Therapeutic target database update 2022: Facilitating drug discovery with enriched comparative data of targeted agents. Nucleic Acids Res..

[43] Stelzer G., Rosen N., Plaschkes I., Zimmerman S., Twik M., Fishilevich S., Stein T.I., Nudel R., Lieder I., Mazor Y. (2016). The GeneCards Suite: From Gene Data Mining to Disease Genome Sequence Analyses. Curr. Protoc. Bioinform..

[44] The GeneCards Suite (2022). Practical Guide to Life Science Databases 27–56. https://www.genecards.org.

[45] Banerjee P., Kemmler E., Dunkel M., Preissner R. (2024). ProTox 3.0: A webserver for the prediction of toxicity of chemicals. Nucleic Acids Res..

[46] Banerjee P., Eckert A.O., Schrey A.K., Preissner R. (2018). ProTox-II: A webserver for the prediction of toxicity of chemicals. Nucleic Acids Res..

[47] Szklarczyk D., Kirsch R., Koutrouli M., Nastou K., Mehryary F., Hachilif R., Gable A.L., Fang T., Doncheva N.T., Pyysalo S. (2023). The STRING database in 2023: Protein-protein association networks and functional enrichment analyses for any sequenced genome of interest. Nucleic Acids Res..

[48] Shannon P., Markiel A., Ozier O., Baliga N.S., Wang J.T., Ramage D., Amin N., Schwikowski B., Ideker T. (2003). Cytoscape: A software environment for integrated models of biomolecular interaction networks. Genome. Res..

[49] Sherman B.T., Hao M., Qiu J., Jiao X., Baseler M.W., Lane H.C., Imamichi T., Chang W. (2022). DAVID: A web server for functional enrichment analysis and functional annotation of gene lists (2021 update). Nucleic Acids Res..

[50] Huang D.W., Sherman B.T., Lempicki R.A. (2009). Systematic and integrative analysis of large gene lists using DAVID bioinformatics resources. Nat. Protoc..

[51] Aleksander S.A., Balhoff J., Carbon S., Cherry J.M., Drabkin H.J., Ebert D., Feuermann M., Gaudet P., Harris N.L., Hill D.P. (2023). The Gene Ontology knowledgebase in 2023. Genetics.

[52] Ashburner M., Ball C.A., Blake J.A., Botstein D., Butler H., Cherry J.M., Davis A.P., Dolinski K., Dwight S.S., Eppig J.T. (2000). Gene ontology: Tool for the unification of biology. The Gene Ontology Consortium. Nat. Genet..

[53] Kanehisa M., Furumichi M., Sato Y., Kawashima M., Ishiguro-Watanabe M. (2023). KEGG for taxonomy-based analysis of pathways and genomes. Nucleic Acids Res..

[54] Morris G.M., Huey R., Olson A.J. (2008). Using AutoDock for ligand-receptor docking. Curr. Protoc. Bioinform..

[55] PyMOL by Schrödinger. http://www.pymol.org/pymol.

[56] Sankar K., Trainor K., Blazer L.L., Adams J.J., Sidhu S.S., Day T., Meiering E., Maier J.K.X. (2022). A Descriptor Set for Quantitative Structure-property Relationship Prediction in Biologics. Mol. Inform..

[57] Tavella D., Ouellette D.R., Garofalo R., Zhu K., Xu J., Oloo E.O., Negron C., Ihnat P.M. (2022). A novel method for in silico assessment of Methionine oxidation risk in monoclonal antibodies: Improvement over the 2-shell model. PLoS ONE.

[58] Sankar K., Krystek S.R., Carl S.M., Day T., Maier J.K.X. (2018). AggScore: Prediction of aggregation-prone regions in proteins based on the distribution of surface patches. Proteins.

[59] Zhu K., Day T., Warshaviak D., Murrett C., Friesner R., Pearlman D. (2014). Antibody structure determination using a combination of homology modeling, energy-based refinement, and loop prediction. Proteins.

[60] Salam N.K., Adzhigirey M., Sherman W., Pearlman D.A. (2014). Structure-based approach to the prediction of disulfide bonds in proteins. Protein Eng. Des. Sel..

[61] Beard H., Cholleti A., Pearlman D., Sherman W., Loving K.A. (2013). Applying physics-based scoring to calculate free energies of binding for single amino acid mutations in protein-protein complexes. PLoS ONE.

[62] Wang L., Zhang T.P., Zhang Y., Bi H.L., Guan X.M., Wang H.X., Wang X., Du J., Xia Y.L., Li H.H. (2016). Protection against doxorubicin-induced myocardial dysfunction in mice by cardiac-specific expression of carboxyl terminus of hsp70-interacting protein. Sci. Rep..

[63] Maillet A., Tan K., Chai X., Sadananda S.N., Mehta A., Ooi J., Hayden M.R., Pouladi M.A., Ghosh S., Shim W. (2016). Modeling Doxorubicin-Induced Cardiotoxicity in Human Pluripotent Stem Cell Derived-Cardiomyocytes. Sci. Rep..

[64] Huang H., Christidi E., Shafaattalab S., Davis M.K., Tibbits G.F., Brunham L.R. (2022). RARG S427L attenuates the DNA repair response to doxorubicin in induced pluripotent stem cell-derived cardiomyocytes. Stem Cell Rep..

[65] Yixin Z., Fenglan J., Xin W., Xiao W., Bo L., Fuchun W., Tao D., Huibo X. (2021). Study on the model of chronic heart failure induced by adriamycin in rats. Chin. J. Gerontol..

[66] Liping T., Fengzhen H., Mingxia X. (2023). Effects of portulaca polysaccharide on atheroscleroti plaque in rats by regulating PPARγ/NFκB pathway. Hebei Med. J..

[67] Kilkenny C., Browne W.J., Cuthi I., Emerson M., Altman D.G. (2012). Improving bioscience research reporting: The ARRIVE guidelines for reporting animal research. Vet. Clin. Pathol..

