# Supplementary material for: DHDK, a Plant-Derived Natural Small Molecule, Protects Against Doxorubicin-Induced Cardiotoxicity via the PPARG-CPT1B-FAO Axis
Source: Pharmaceuticals (Basel). 2025 Nov 18;18(11):1759. doi: 10.3390/ph18111759 (PMC12655265; doi:10.3390/ph18111759)
Supplement: Supplementary file 1 [file pharmaceuticals-18-01759-s001.zip › pharmaceuticals-3930019-supplementary.pdf]

pharmaceuticals

Supplementary Materials: DHDK, a Plant-Derived Natural Small Molecule, Protects Against Doxorubicin-Induced Cardiotoxicity via the PPARG-CPT1B-FAO Axis

Jing Hong, Fangyu Zhang, Ruizhen Zhang, Hongyang Fu, Dongang Shen, Xinyue Wang, Yuting Yang, Jiamei Wu, Lin Meng, Hongyang Lü, Xiwei Jiang, Yunli Zhao

1. DHDK characterization using in this paper

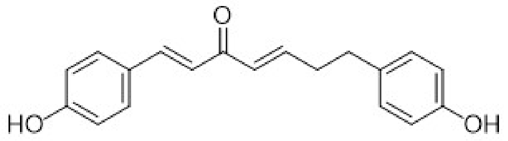

Figure S1. The structure of DHDK.

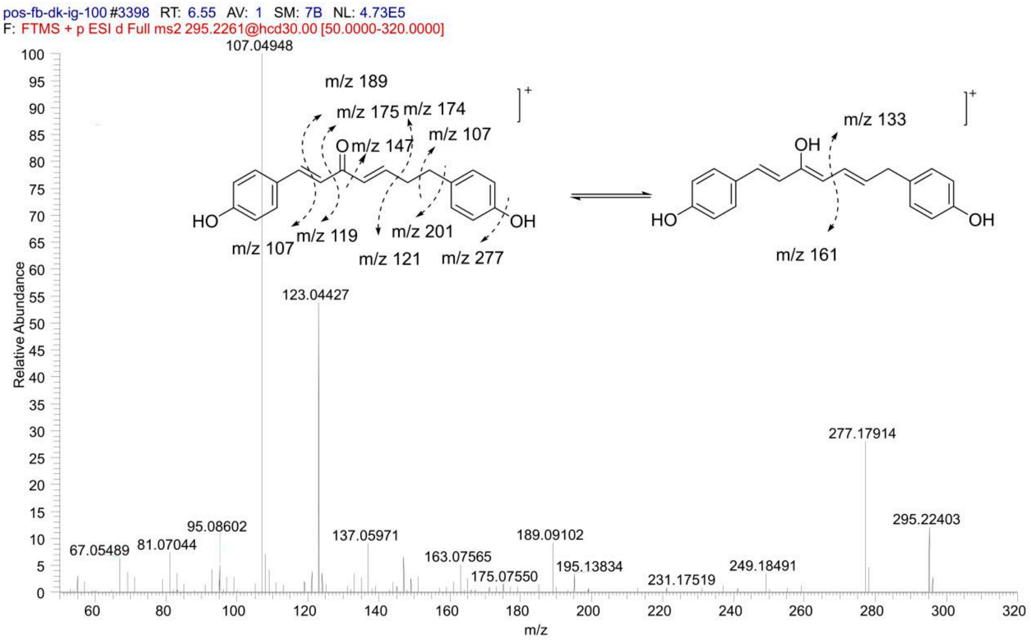

Figure S2. The MS spectrum of DHDK (positive ion).

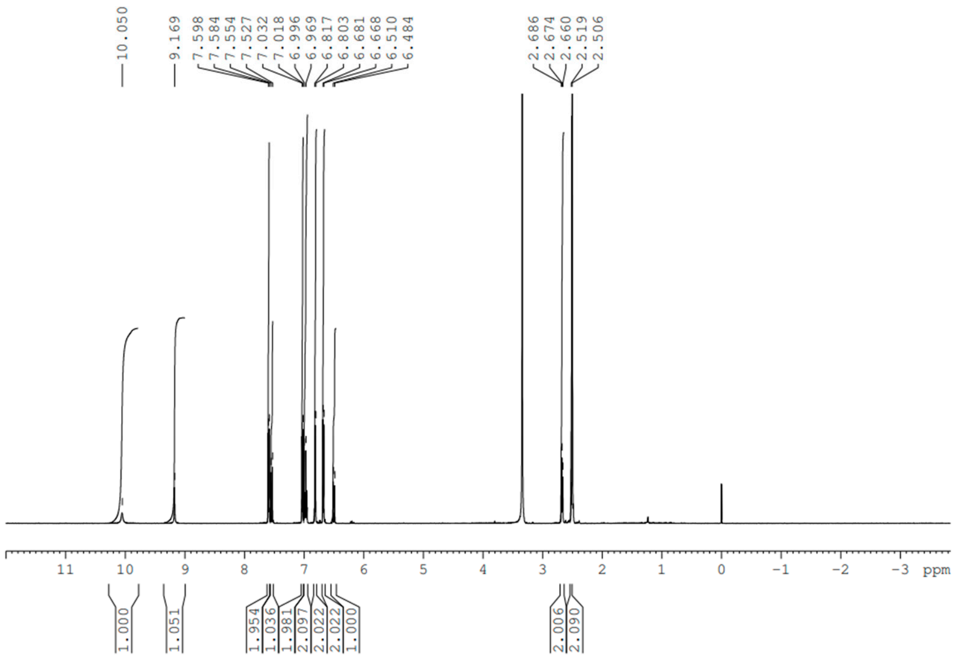

^{1}H NMR (600 MHz, d_6-DMSO) δ 10.05 (s, 1H), 9.17 (s, 1H), 7.59 (d, *J* = 8.58 Hz, 2H), 7.54 (d, *J* = 15.99 Hz, 1H), 7.03 (d, *J* = 8.32 Hz, 2H), 7.00-6.94 (m, 2H), 6.81 (d, *J* = 8.62 Hz, 2H), 6.67 (d, *J* = 8.28 Hz, 2H), 6.50 (d, *J* = 15.6 Hz, 1H), 2.67 (t, *J* = 7.29 Hz, 2H), 2.54-2.50 (m, 2H)

Figure S3. The NMR(H) spectrum of DHDK.

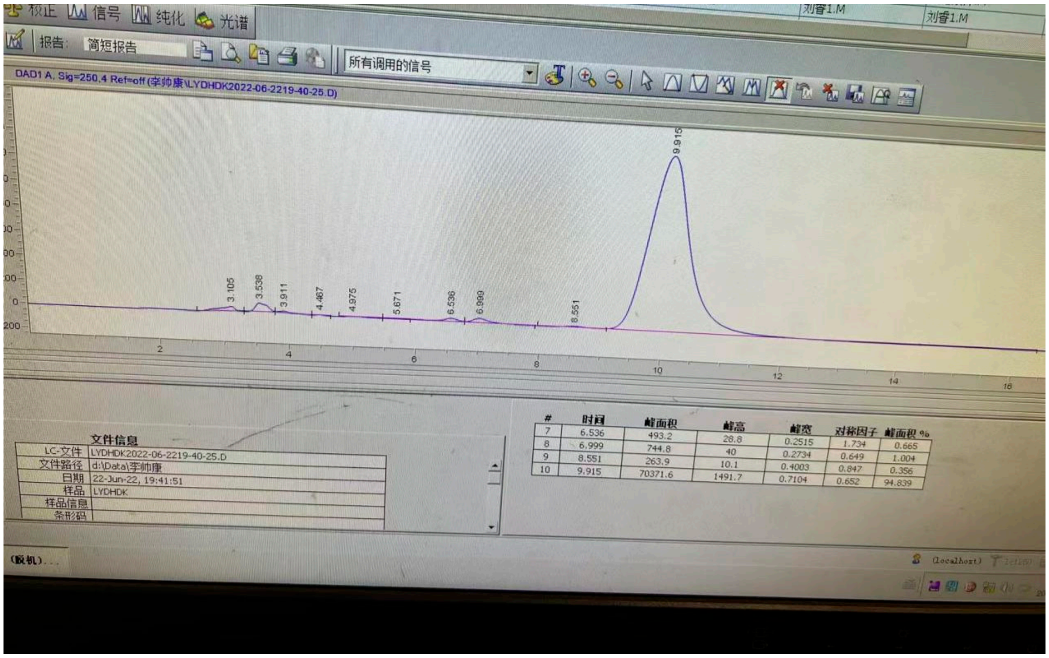

Figure S4. The purity of DHDK using HPLC.

2. Below are the previously reported [34] DHDK Characterization

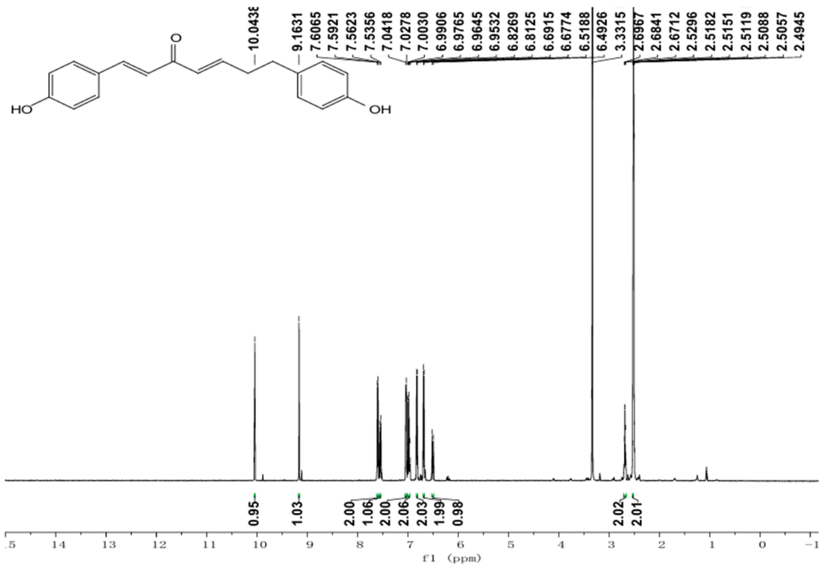

Figure S5. The NMR(H) spectrum of previouly reported DHDK.

^{1}H NMR (600 MHz, d_6-DMSO) δ 10.04 (s, 1H), 9.16 (s, 1H), 7.60 (d, *J* = 8.6 Hz, 2H), 7.55 (d, *J* = 16.0 Hz, 1H), 7.03 (d, *J* = 8.4 Hz, 2H), 7.00-6.95 (m, 2H), 6.82 (d, *J* = 8.6 Hz, 2H), 6.68 (d, *J* = 8.4 Hz, 2H), 6.51 (d, *J* = 15.7 Hz, 1H), 2.68 (t, *J* = 7.7 Hz, 2H), 2.53-2.49 (m, 2H).

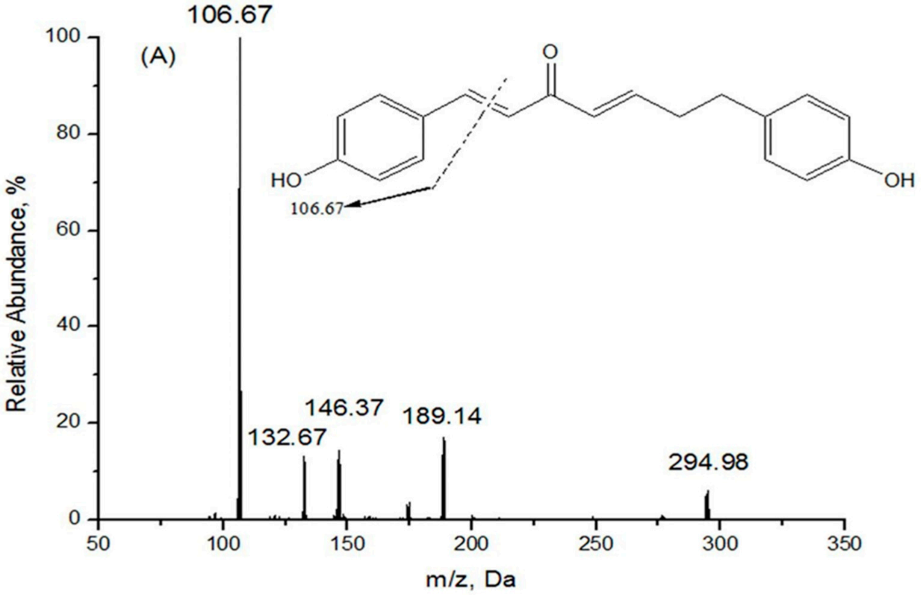

Figure S6. The MS spectrum of previouly reported DHDK.

Table S1. ProTox-3.0–Prediction of TOXicity of chemicals.

ProTox-3.0 - Prediction of TOXicity of chemicals

Classification	Target	Shorthand	Prediction	Probability
Organ toxicity	Hepatotoxicity	dili	Inactive	0.86
Organ toxicity	Neurotoxicity	neuro	Active	0.74
Organ toxicity	Nephrotoxicity	nephro	Active	0.80
Organ toxicity	Respiratory toxicity	respi	Active	0.91
Organ toxicity	Cardiotoxicity	cardio	Active	0.64
Toxicity end points	Carcinogenicity	carcino	Inactive	0.90
Toxicity end points	Immunotoxicity	immuno	Active	0.99
Toxicity end points	Mutagenicity	mutagen	Active	0.98
Toxicity end points	Cytotoxicity	cyto	Active	0.94
Toxicity end points	BBB-barrier	bbb	Inactive	1.0
Toxicity end points	Ecotoxicity	eco	Inactive	0.58
Toxicity end points	Clinical toxicity	clinical	Active	0.84
Toxicity end points	Nutritional toxicity	nutri	Inactive	0.69
Tox21-Nuclear receptor signalling pathways	Aryl hydrocarbon Receptor (AhR)	nr_ahr	Inactive	0.92
Tox21-Nuclear receptor signalling pathways	Androgen Receptor (AR)	nr_ar	Inactive	0.99
Tox21-Nuclear receptor signalling pathways	Androgen Receptor Ligand Binding Domain (AR-LBD)	nr_ar_lbd	Inactive	0.55
Tox21-Nuclear receptor signalling pathways	Aromatase	nr_aromatase	Active	0.52
Tox21-Nuclear receptor signalling pathways	Estrogen Receptor Alpha (ER)	nr_er	Inactive	0.73
Tox21-Nuclear receptor signalling pathways	Estrogen Receptor Ligand Binding Domain (ER-LBD)	nr_er_lbd	Inactive	0.74
Tox21-Nuclear receptor signalling pathways	Peroxisome Proliferator Activated Receptor Gamma (PPAR-Gamma)	nr_ppar_gamma	Inactive	0.97
Tox21-Stress response pathways	Nuclear factor (erythroid-derived 2)-like 2/ antioxidant responsive element (nrf2/ARE)	sr_are	Inactive	0.98
Tox21-Stress response pathways	Heat shock factor response element (HSE)	sr_hse	Inactive	0.98
Tox21-Stress response pathways	Mitochondrial Membrane Potential (MMP)	sr_mmp	Inactive	0.56
Tox21-Stress response pathways	Phosphoprotein (Tumor Supressor) p53	sr_p53	Active	0.52
Tox21-Stress response pathways	ATPase family AAA domain-containing protein 5 (ATAD5)	sr_atad5	Inactive	0.63
Molecular Initiating Events	Thyroid hormone receptor alpha (THRα)	mie_thr_alpha	Inactive	0.90
Molecular Initiating Events	Thyroid hormone receptor beta (THRβ)	mie_thr_beta	Inactive	0.78
Molecular Initiating Events	Transtyretrin (TTR)	mie_ttr	Inactive	0.97
Molecular Initiating Events	Ryanodine receptor (RYR)	mie_ryr	Inactive	0.98
Molecular Initiating Events	GABA receptor (GABAR)	mie_gabar	Inactive	0.96
Molecular Initiating Events	Glutamate N-methyl-D-aspartate receptor (NMDAR)	mie_nmdar	Inactive	0.92
Molecular Initiating	alpha-amino-3-hydroxy-5-methyl-4-	mie_ampar	Inactive	0.97

Classification	Target	Shorthand	Prediction	Probability
Events	isoxazolepropionate receptor (AMPAR)			
Molecular Initiating Events	Kainate receptor (KAR)	mie_kar	Inactive	0.99
Molecular Initiating Events	Achetylcholinesterase (AChE)	mie_ache	Inactive	0.86
Molecular Initiating Events	Constitutive androstane receptor (CAR)	mie_car	Inactive	0.98
Molecular Initiating Events	Pregnane X receptor (PXR)	mie_pxr	Inactive	0.92
Molecular Initiating Events	NADH-quinone oxidoreductase (NADHOX)	mie_nadhox	Inactive	0.97
Molecular Initiating Events	Voltage gated sodium channel (VGSC)	mie_vgsc	Inactive	0.95
Molecular Initiating Events	Na+/I- symporter (NIS)	mie_nis	Inactive	0.98
Metabolism	Cytochrome CYP1A2	CYP1A2	Inactive	0.99
Metabolism	Cytochrome CYP2C19	CYP2C19	Inactive	0.97
Metabolism	Cytochrome CYP2C9	CYP2C9	Inactive	0.73
Metabolism	Cytochrome CYP2D6	CYP2D6	Inactive	0.92
Metabolism	Cytochrome CYP3A4	CYP3A4	Inactive	0.98
Metabolism	Cytochrome CYP2E1	CYP2E1	Inactive	0.99

Table S2. Lipidomics pathway enrichment results.

Pathway name	Pathway lipids
Linoleic acid metabolism	25
Glycine, serine and threonine metabolism	3
Ether lipid metabolism	16
Glycosylphosphatidylinositol (GPI)-anchor biosynthesis	3
Sphingolipid metabolism	21
alpha-Linolenic acid metabolism	23
Glycerophospholipid metabolism	26
Inositol phosphate metabolism	9
Sphingolipid signaling pathway	9
Arachidonic acid metabolism	75
Autophagy - other	3
Autophagy - animal	4
Necroptosis	4
Phosphatidylinositol signaling system	11
Ferroptosis	11
Neurotrophin signaling pathway	3
Adipocytokine signaling pathway	3
Retrograde endocannabinoid signaling	8
Insulin resistance	4
AGE-RAGE signaling pathway in diabetic complications	2
Pathogenic Escherichia coli infection	1
Tuberculosis	5
Leishmaniasis	4
Kaposi's sarcoma-associated herpesvirus infection	3
Choline metabolism in cancer	5
Systemic lupus erythematosus	1
Amoebiasis	4

Table S3. List of comparative molecular docking with native ligand and DHDK.

	PDB number	Native ligand	Docking (kcal/mol) with native ligand	RMSD (Å)	Docking (kcal/mol) with DHDK(drug)	RMSD (Å)
ALB	1BKE	B3I	−7.65	0	−8.398	2.294
CASP3	1GFW	MSI	−5.41	0.994	−7.541<br>−4.323*	1.526<br>2.042*
PPARG	1FM6	BRL	−7.12	2.264	−9.745<br>−7.495*	2.581<br>1.824*
IGF1	1GZR	C15	−3.58	2.772	−6.081	0.957
HSP90AA1	1BYQ	ADP	−10.78	1.57	−7.058	1.919
ESR1	1A52	EST	−9.85	0.001	−6.697	3.767
SRC	1A07	DIP	−6.56	2.376	−6.219	1.551

*Covalent molecular docking

Table S4. List of siRNA(CPT1B) sequence.

	Forward	Reverse
siCPT1B	UCGAAUAAGGCGUUUCUUCCA	GAAGAAACGCCUUAUUCGAAU

Table S5. List of sequence and other information of primers used in quantitative real-time PCR.

Gene symbol	Primer sequence	Amplicon size (bp)	Blast result
PPARG	TCCCGTTCACAAGAGCTGAC<br>ATAATAAGGCGGGGACGCAG	107	NM_001145367.1
FABP3	CAGGTGGCTAGCATGACCAA<br>CAGTGTCACGACCGACTTGA	168	NM_024162.2
PDK4	TCCTTCACACCTTCACCACA<br>AAAGAGGCGGTCAGTAATCC	190	NM_053551.2
CPT1B	AGCCCCATCATGGTGAACAG<br>CCAGTTTGCGGCGATACATG	124	NM_013200.2
ACADVL	GGAGCTTTGGAGGGGTTACC<br>GGCCCATCTTCTTTTCGGGGA	89	XM_063268458.1
β-actin	CATCCGTAAAGACCTCTATGCCAAC<br>ATGGAGCCACCGATCCACA	171	NM_031144.3
